# Akt/mTOR integrate energy metabolism with Wnt signal to influence wound epithelium growth in *Gekko Japonicus*

**DOI:** 10.1038/s42003-022-04004-5

**Published:** 2022-09-27

**Authors:** Qinghua Wang, Zuming Mao, Zhuang Liu, Man Xu, Shuai Huang, Yin Wang, Yanran Xu, Longju Qi, Mei Liu, Yan Liu

**Affiliations:** 1 Key Laboratory of Neuroregeneration of Jiangsu Province and Ministry of Education, Co-innovation Center of Neuroregeneration, Nantong University, Nantong, 226001 China; 2 Comparative Medicine Research Institution, Nantong University, Nantong, 226001 China; 3 Affiliated Nantong Hospital 3 of Nantong University, Nantong University, Nantong, 226001 China

**Keywords:** Herpetology, Regeneration

## Abstract

The formation of wound epithelium initiates regeneration of amputated tail in *Gekko japonicus*. Energy metabolism is indispensable for the growth of living creatures and typically influenced by temperature. In this study, we reveal that low temperature lowers energy metabolism level and inhibits the regeneration of amputated tails of *Gekko japonicus*. We further find that low temperature attenuates the activation of protein kinase B (Akt) and mammalian target of rapamycin (mTOR) in regenerated tissues upon injury signals, and the inhibition of Akt hinders proliferation of the wound epithelium. Additionally, wingless/integrated (Wnt) inhibition suppresses epithelium proliferation and formation by inhibiting Akt activation. Finally, low temperature elevates the activity of adenylate-activated kinase (AMPK) pathway and in turn attenuates wound epithelium formation. Meanwhile, either mTOR downregulation or AMPK upregulation is associated with worse wound epithelium formation. Summarily, low temperature restricts wound epithelium formation by influencing energy sensory pathways including Akt/mTOR and AMPK signaling, which is also modulated by injury induced Wnt signal. Our results provide a mechanism that incorporates the injury signals with metabolic pathway to facilitate regeneration.

## Abstract (web summary)

Low temperature inhibits the regeneration of amputated tails of Gekko japonicus by influencing the energy sensory Akt/mTOR pathway, which is also modulated by injury-induced Wnt signal.

## Introduction

Regenerative capacity varies among different species. In mammals, limited regeneration capacity has been observed in skin, epithelial mucosa of digestive system, and liver. However, lower vertebrates show higher innate regenerative abilities, where complete regeneration of tissues and organs can be achieved and preserved throughout life. For example, salamanders regenerate limbs^1^, zebrafishes regenerate their hearts^2^, and lizards regenerate their tails^3^. Similarly, *Gekko japonicus* has a strong regeneration ability to regenerate its severed tail^4^. The injured site forms wound epithelium, and then develops a bud that grows and differentiates into skin, muscle, cartilage and neural tissues^4,5^. Finally, the newborn tail shows morphological regeneration and functional recovery. Therefore, *Gekko japonicus* is a feasible animal model for regeneration research.

Tissue regeneration is an energy-intensive process. However, the role and mechanism of the metabolic pathways in regulating appendage regeneration remain elusive. We found that the regeneration ability of *Gekko japonicus* was severely inhibited when the surrounding temperature was below 10 °C. This effect was consistent with that reported by Milsom et. al, who proposed the metabolic level was an essential criterion for successful regeneration^6^. Then, we proposed that the low temperature treatment could lead to a state of low metabolic rate and failure of tail regeneration. This model could provide an alternative opportunity to investigate how the metabolic pathway to regulate regeneration process.

Several studies have shown that the protein kinase B/mammalian target of rapamycin (Akt/mTOR) and adenylate-activated kinase (AMPK) pathways are important metabolic regulatory pathways for energy sensing^7–9^. In addition, phosphoinositide 3 kinase (PI3K) and phosphatase and tensin homologous protein (PTEN) are important modulator of Akt/mTOR pathway^10,11^. mTOR, as a serine/threonine protein kinase, plays an important role in sensing nutritional level of cells and regulating metabolism and cell behavior including protein synthesis, cell growth, movement and proliferation^12–14^. AMPK is a kind of conserved serine/threonine protein kinase that is very sensitive to cell energy state, as indicated by the ratio of AMP and ATP^10,15^. However, the regulatory mechanism of the energy sensing pathways in lizard tail regeneration remains unclear.

The initial stage of gecko’s tail regeneration was mainly composed of two courses, one was wound epithelium covering and the other was blastema formation^4,5,16^. The wound epithelium gradually became thicker and formed apical epithelia (AEC), which was similar in structure and function to the apical ectodermal ridge (AER) during limb bud development^17,18^. The injury-induced signals triggered formation of AEC, meanwhile the AEC could secrete many growth factors to facilitate the proliferation and migration of other cells and orchestrate the regeneration process. The Wnt pathway is a well-recognized signal to regulate the initiation of regeneration in many tissues^19–22^.

However, whether and how the Wnt signal modulates the metabolic events during regeneration of tail regeneration of lizard is unclear yet. Here, low temperature was found to hinder the formation of wound epithelium, which might due to activation of AMPK pathway. Then, AMPK induced inhibition of Akt/mTOR decreased the proliferation of wound epithelium. Furthermore, we revealed that Wnt pathway activation promoted Akt/mTOR activity and thus facilitated wound epithelium formation. Results above shed light on the interaction between the injury signals and metabolic pathway during regeneration.

## Results

### Metabolic impedance caused by low temperature constrains blastema growth

**Fig. 1:**
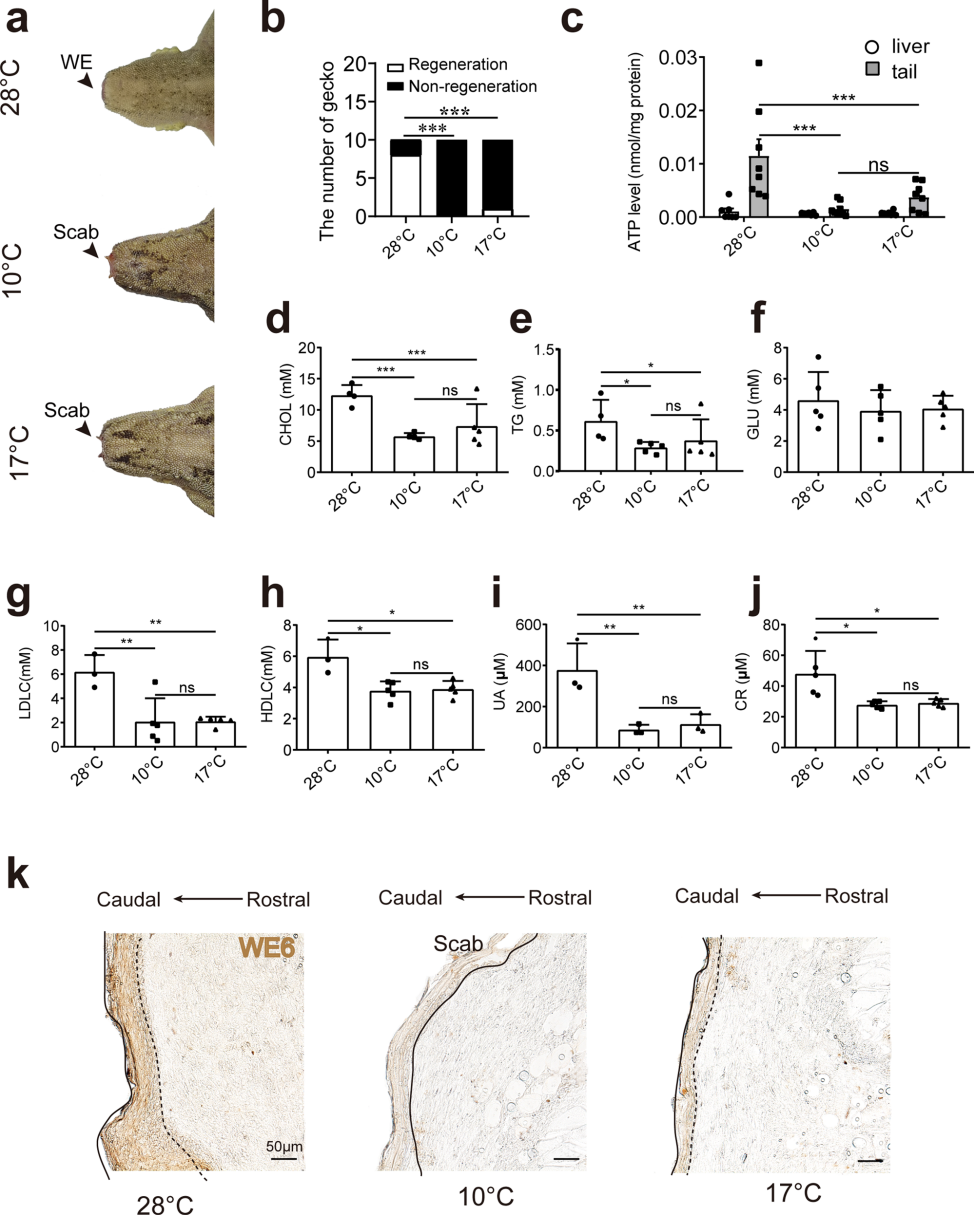
Energy homeostasis was altered in the non-regeneration geckos. **a** Wound epithelium (WE) formation in geckos in high (28 °C) and low temperature groups (17 °C and 10 °C). **b** Number of regenerated geckos in high (28 °C) and low temperature groups (17 °C and 10 °C), *N* = 10 for each group. **c** ATP levels in liver and tail tissue in geckos from high (28 °C) and low temperature groups (17 °C and 10 °C), *N* = 7–8. **d**–**j** Serum glucose (GLU), cholesterol (CHOL), triglyceride (TG), low-density lipoprotein cholesterol (LDLC), high-density lipoprotein cholesterol (HDLC), uric acid (UA), and creatinine (CR) concentration, *N* = 4–5. **k** Immunohistochemical analysis of WE6 (marker of wound epithelium) staining in control and low temperature groups (17 °C and 10 °C, respectively). Solid line indicates the caudal border of wound epithelium, dotted line indicates border between wound epithelium and blastema. Scale bar = 50 µm. Values represent means ± SD, ^ns^*p* > 0.05, *p < 0.05, **p < 0.01, ***p < 0.001.

The innate ability in regeneration of amputated tail was based on the formation of wound epithelium and blastema in geckos^5^. Wild geckos experience temperature and metabolic variance during different seasons^6,23^. Then, we asked whether low temperature affect regeneration process. We tested the tail regeneration at 28 °C, 10 °C and 17 °C respectively to investigate the effect. In 28 °C group, 80% of the geckos successfully initiated regeneration, while none and 3% geckos could initiate regeneration in 10 °C group and 17 °C group respectively (Fig. 1a, b). Then, we termed 28 °C group as normal group, 10 °C group and 17 °C group as low temperature groups. To testify the metabolic variances in groups of different temperature, the tissues of the geckos were collected and examined. ATP levels were significantly higher in the tail tissues from the 28 °C group compared to that from the 10 °C group and 17 °C group (Fig. 1c). However, no significant difference was observed in the livers of normal and low temperature groups (Fig. 1c). To understand this difference, metabolic characteristics, such as cholesterol (CHOL, Fig. 1d), triglycerol (TG, Fig. 1e), serum glucose (GLU, Fig. 1f), low-density lipoprotein cholesterol (LDLC, Fig. 1g), high-density lipoprotein cholesterol (HDLC, Fig. 1h), uric acid (UA, Fig. 1i), and Creatinine (CR, Fig. 1j), was analyzed to determine the levels of glucose, lipid and protein metabolism. These results revealed that low temperature decreased the systematic metabolism. In addition, the formation of wound epithelium that positively stained with WE6 antibody was observed in low temperature and control group. We found that the wound epithelium formation was hindered in the 10 °C group and 17 °C group in a temperature dependent manner (Fig. 1k).

### Akt/mTOR pathway activation is inhibited during tail regeneration under low temperature

**Fig. 2:**
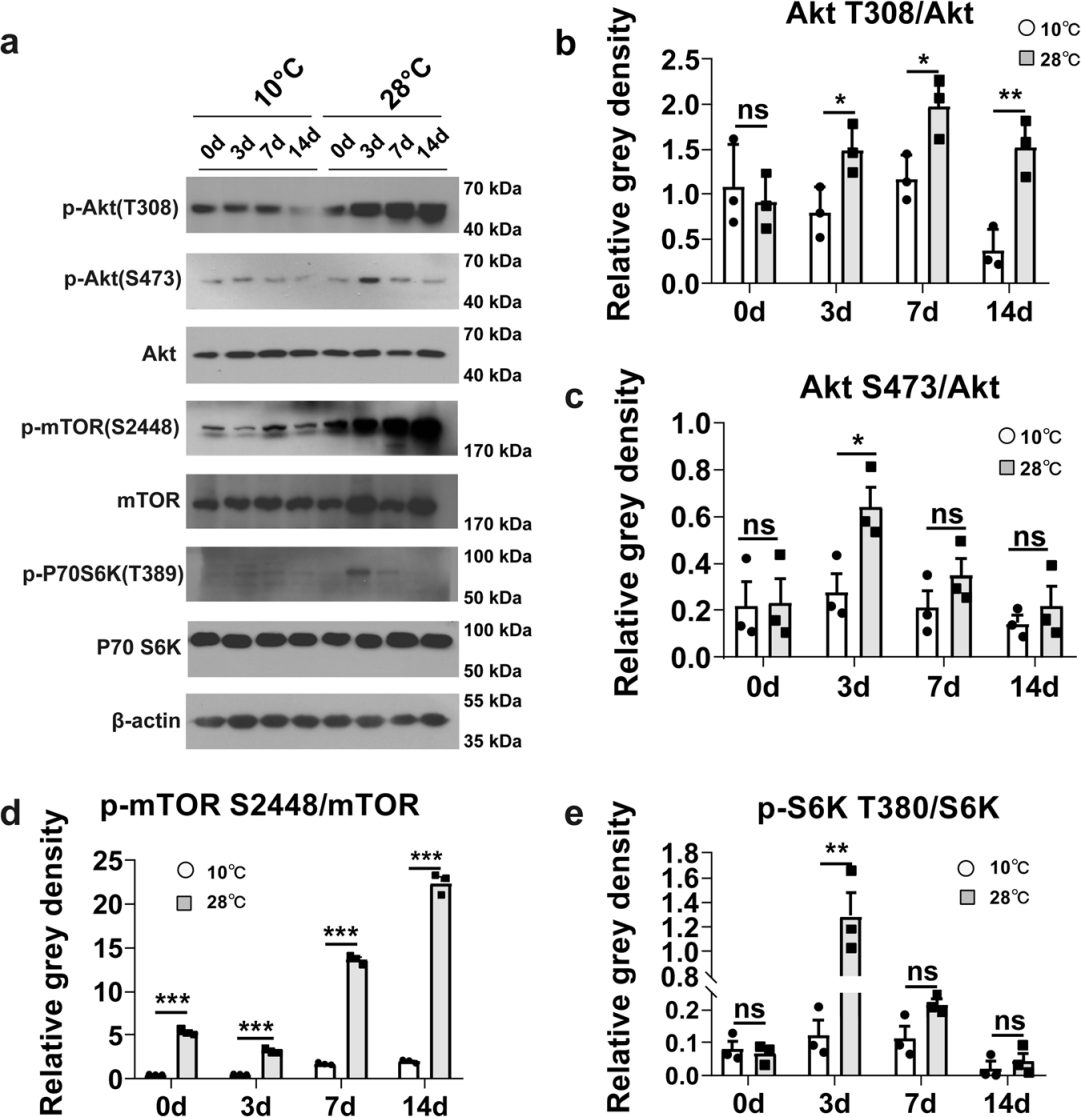
Akt/mTOR pathway was involved in the regeneration process. **a** Protein expression of Akt, p-Akt (Ser473, Thr308), p-mTOR (Ser2448), mTOR, p-P70 S6K (Thr389), P70 S6K in the high and low temperature groups on days 0, 3, 7 and 14. **b**–**e** Relative expression of p-Akt (Thr308)/Akt, p-Akt (Ser473)/Akt, p-P70 S6K (Thr389), p-mTOR (Ser2448)/mTOR, p-P70 S6K (Thr389)/P70 S6K in Fig. 2a. Values represent mean ± SD, *N* = 3, ^ns^*p* > 0.05, *p < 0.05, **p < 0.01, ***p < 0.001.

A thin layer of wound epithelium appeared on the 7th day after tail amputation, which induced the blastama formation thereafter^24^. Akt and mTOR are known to be involved in the regulation of growth, development and energy utilization^25,26^. Therefore, the activity of Akt and mTOR were analyzed in the tail tissues at day 0, 3, 7 and 14 after injury in the low and normal temperature groups. The Akt activity is indicated by phosphorylation of Thr308 and Ser473^27^. Our results revealed that Akt activation level in the normal temperature (28 °C) group was significantly augmented at the beginning stage of regeneration. The phosphorylation of Thr308 was enhanced on day 3 (Fig. 2a, b), day 7 (Fig. 2a, b) and day 14 (Fig. 2a, b), and a significant increase of Ser473 phosphorylation was also observed on day 3 (Fig. 2a, c). Meanwhile, we evaluated the mTOR activity by phosphorylation on Ser2448 that usually infers activation of mTORC1^28^. The results proposed that mTOR activity was higher in tail tissues from 28 °C group than 10 °C group on day 0 (Fig. 2a, d), day 3 (Fig. 2a, d), day 7 (Fig. 2a, d). Furthermore, we detected the activity of P70 S6K, a downstream target of mTOR that is required for cell growth and cell cycle progression^29,30^. The level of p-P70 S6K (Thr389) were significantly enhanced in geckos from normal temperature group on day 3 (Fig. 2a, e). In conclusion, the Akt, mTOR and P70 S6K activities were boosted in normal group during the early stage of blastama formation.

### Inhibition of Akt lowers the proliferation of wound epithelium

**Fig. 3:**
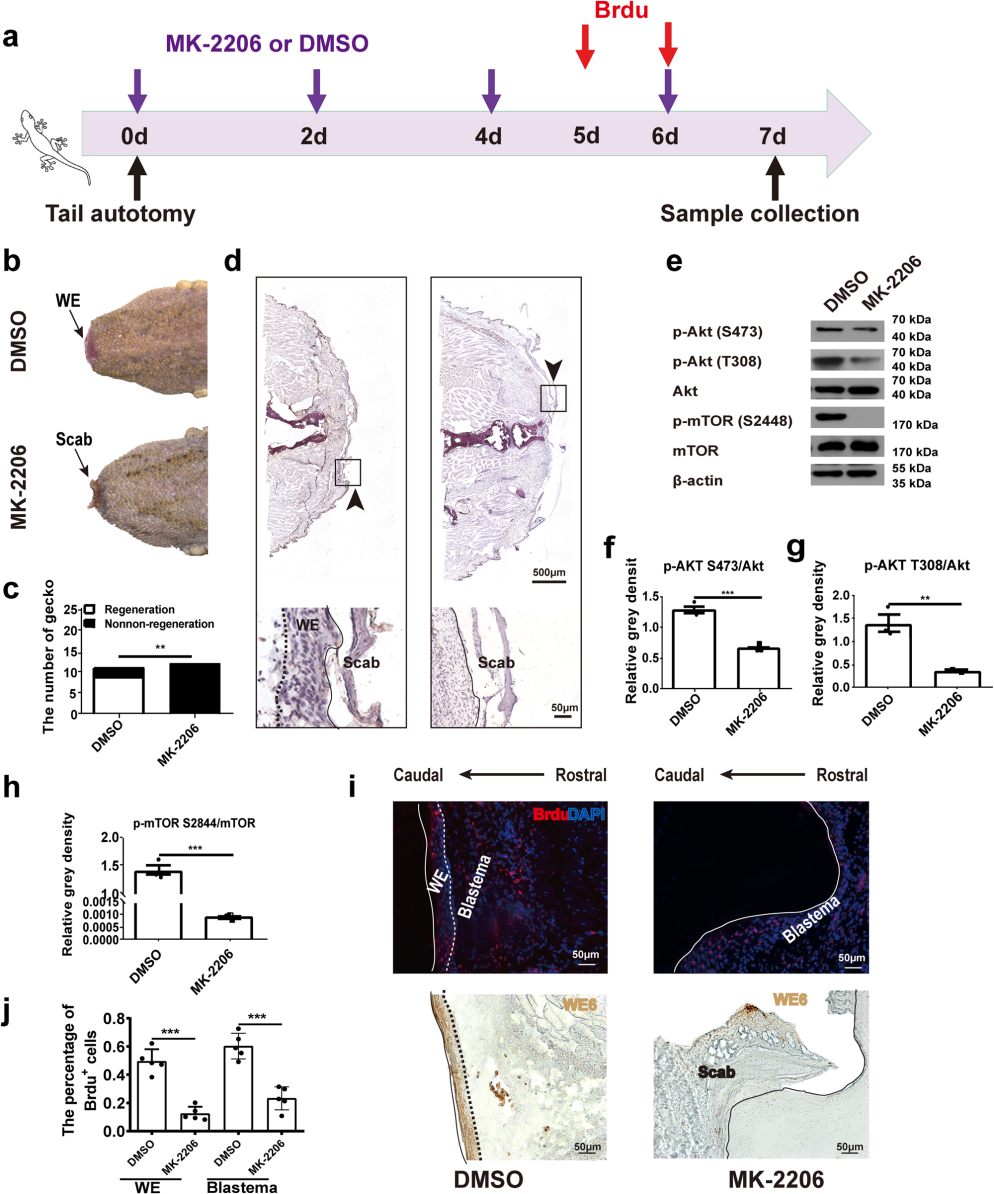
Akt blocking lowered the proliferation of wound epithelium. **a** The schematic picture of timeline for drug delivering and sample collection. **b** Formation of wound epithelium (WE) in geckos in the control (DMSO) and Akt inhibition groups (MK-2206). **c** Number of geckos with regeneration in control and Akt inhibition groups. *N* = 10 for each group. **d** H&E staining of tail tissues from control and Akt inhibition groups. Solid arrow indicates the injury site, dashed box indicates the magnified area, dotted line indicates border between wound epithelium and scab, and solid line indicates junction between rostral end of wound epithelium and blastema. Scale bar = 500 µm or 50 µm. *N* = 3. **e** Protein levels of p-Akt (Ser473, Thr308), Akt, p-mTOR (Ser2448), and mTOR in tail tissues. **f**–**h** Relative level of p-Akt (Ser473)/Akt, p-Akt (Thr308)/Akt, and p-mTOR (Ser2448)/mTOR in Fig. 3d. *N* = 3. **i** Immunofluorescence analysis of BrdU staining in control (DMSO) and Akt inhibition group (MK-2206) (upper panel). Immunohistochemistry analysis of WE6 staining of the same location of the samples. Solid line indicates the caudal border of wound epithelium, dotted line indicates border between wound epithelium and blastema. Scale bar = 50 µm. **j** Number of BrdU positive cells in control and Akt inhibition group. Values represent mean ± SD, *N* = 5, ^ns^*p* > 0.05, *p < 0.05, **p < 0.01, ***p < 0.001.

To investigate the effect of Akt activity on tail regeneration, MK-2206, a Akt inhibitor^31^ was injected intraperitoneally (i.p.) in tail-amputated geckos followed timeline listed Fig. 3a. As an important indicator of regeneration initiation, the formation of wound epithelium was checked at day 7 after the surgery. As illustrated in Fig. 3b, c, regeneration was clearly suppressed in the Akt inhibition group. Geckos in the control group shed the scab and covered the injured site with smooth wound epithelium, while those in inhibition group were still wrapped with scab. Similar results were also observed in the morphological examination that revealed only scab and no wound epithelium in the inhibition group (Fig. 3d). Following confirmation of the morphological changes, the newborn tissues were collected and analyzed by western blotting, which showed that phosphorylation level of Akt Ser473 (Fig. 3e, f) and Akt Thr308 (Fig. 3e, g) were significantly decreased. In addition, a significant difference was also observed in the phosphorylation of mTOR Ser2448 (Fig. 3e, h), whereas no significant difference was detected in total Akt and mTOR. WE6 is a common marker of wound epithelium and BrdU incorporation is an efficient marker of cell proliferation. The BrdU positive cells were significantly decreased in the Akt inhibition group compared to that in the control group (Fig. 3i, j). These data suggest that Akt activation is essential for proliferation of blastema cell and formation of wound epithelium, ultimately affecting the regeneration of the injured tails.

### Wnt/β-catenin signal regulates wound epithelium formation and Akt activity

**Fig. 4:**
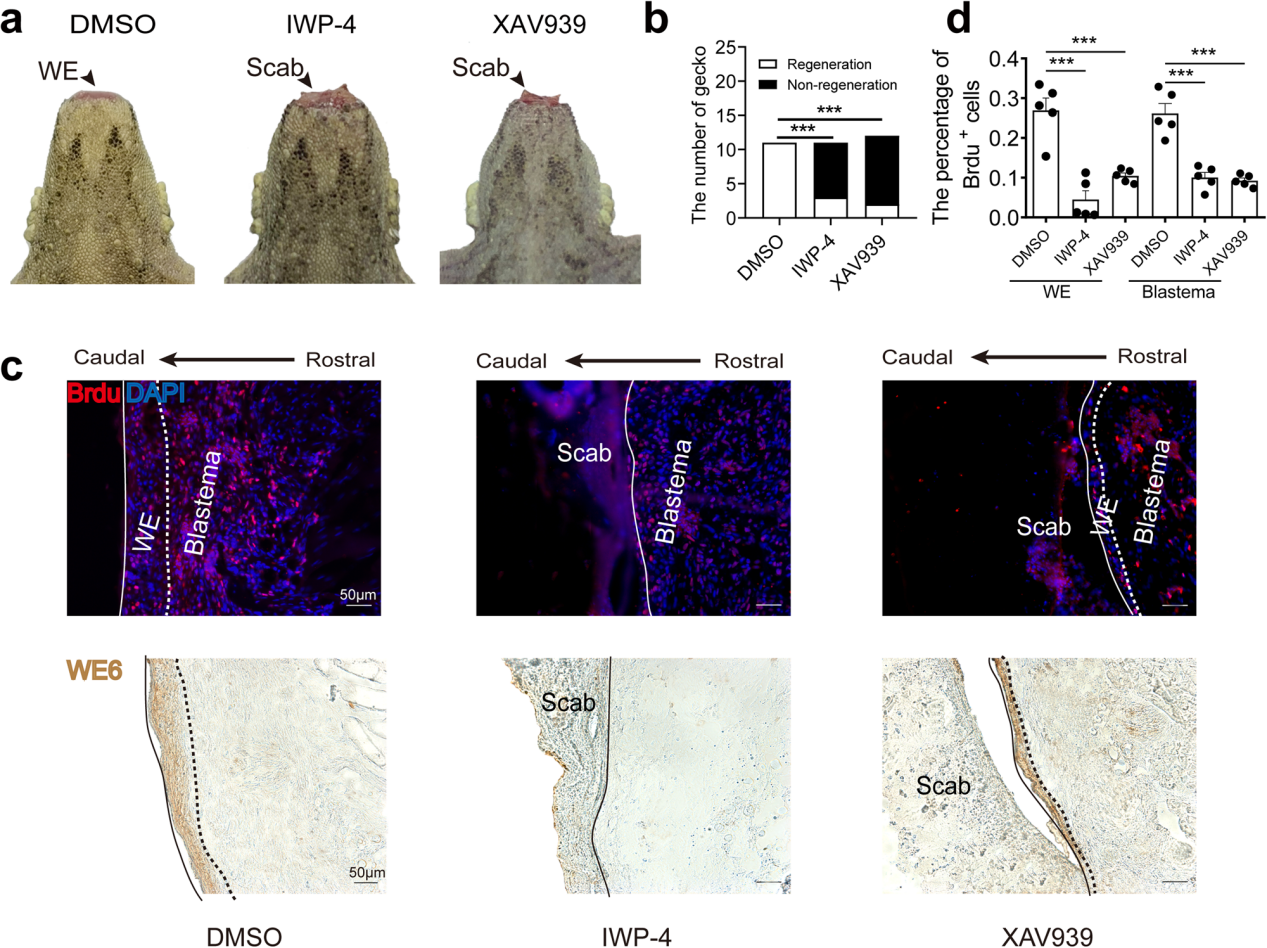
Wnt inhibition decreased the proliferation of wound epithelium. **a** Formation of wound epithelium (WE) in control (DMSO) and Wnt inhibition groups (IWP-4 and XAV939). **b** Number of geckos with regeneration in control and Wnt inhibition groups. *N* = 10. **c** Immunofluorescence analysis of BrdU staining in control and Wnt inhibition groups (upper panel). Immunohistochemical analysis of WE6 staining in the same location of the samples. Solid line indicates the caudal border of wound epithelium, dotted line indicates border between wound epithelium and blastema. Scale bar = 50 µm. **d** Number of BrdU positive cells in control and Wnt inhibition groups. Values represent mean ± SD, *N* = 5, ^ns^*p* > 0.05, *p < 0.05, **p < 0.01, ***p < 0.001.

The Wnt/β-catenin axis is pivotal in appendage regeneration^19,24^. We investigated whether the Wnt inhibitors, IWP-4 and XAV939, affect the formation of wound epithelium and tail regeneration. The formation of wound epithelium was examined 7 days after surgery. Both, the formation of wound epithelium (Fig. 4a) and rate of regeneration (Fig. 4b) were clearly affected by IWP-4 or XAV939 treatment. Morphological examination showed the presence of ample amount of scab tissue, instead of wound epithelium, bundled at the injury site (Fig. 4c, lower panel). Cell proliferation was analyzed using BrdU staining, and WE6 served as a marker to outline the location of the wound epithelium. The results revealed that Wnt inhibition suppressed cell proliferation (Fig. 4c, upper panel, and 4d).

**Fig. 5:**
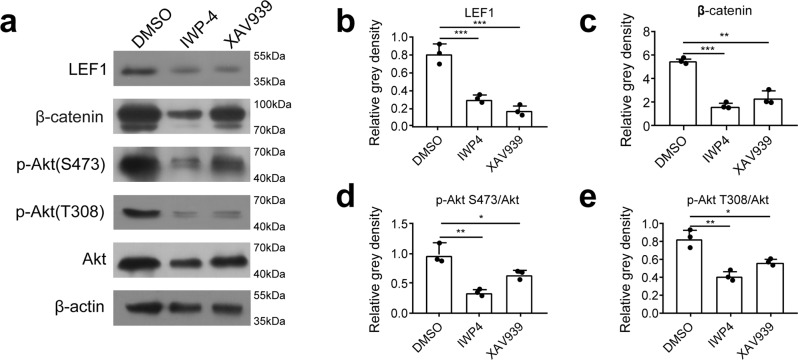
Akt phosphorylation was inhibited by Wnt antagonist during tail regeneration. **a** Protein content of LEF1, β-Catenin and p-Akt (Ser473, Thr308) in tail tissues from Wnt inhibition groups (IWP-4 and XAV939) were significantly reduced compared with that control (DMSO) group. **b**–**e** Relative level of proteins in Fig. 5a. Values represent mean ± SD, *N* = 3, ^ns^*p* > 0.05, *p < 0.05, **p < 0.01, ***p < 0.001.

We have revealed that both low temperature or inhibitor induced Akt inhibition impaired the epithelium formation, and the Wnt signals were also implicated in the proliferation of epithelium cells. Then, we question whether Wnt signal regulates Akt activity during tail regeneration. The Wnt inhibitor, IWP-4 and XAV939 were applied in the experiments. We detected the effect of Wnt inhibitor by evaluating the level in β-Catenin and lymphoid enhancer-binding factor 1 (LEF1) in samples. LEF1, an effector transcription factor of Wnt pathway activation, plays an important role in regulating cell proliferation, differentiation and apoptosis. IWP-4 and XAV939 efficiently decreased the level of LEF1 (Fig. 5a, b), β-catenin (Fig. 5a, c), which indicated that the two inhibitors might negatively regulate β-catenin dependent Wnt pathway. We further investigated whether Wnt blocking results in Akt inhibition. The results showed that the phosphorylation of Akt were both inhibited at the sites of Serine 473 (Fig. 5a, d) and Threonine 308 (Fig. 5a, e). These findings implied that Akt activity was regulated by Wnt/β-catenin during regeneration. Then, we asked whether Wnt signal directly influence Akt activity. Previous study on the effect of Wnt signal on appendage regeneration revealed that Wnt signal could induce the expression of fibroblast growth factors^19^, which facilitated the proliferation of blastema cells. We tested this effect of Wnt agonist (SKL2001 or CHIR) in cultured blastema cells, and found the Akt phosphorylation was augmented (Supplementary Fig. 1a–c). Meanwhile, we also observed that FGF8, FGF10 and FGF20 were up-regulated upon treatment of SKL2001, while inhibition of Akt activity attenuated the upregulation of FGFs (Supplementary Fig. 1d), which is consistent with the hypothesis as other reports. These factors might contribute to the Akt activity.

We further investigated whether Wnt agonist could overcome the low temperature-induced deficiency in tail regeneration. We detected the effect of Wnt agonist on the formation of WE6 positive epithelium at low temperature. Compared with geckos under normal temperature (Supplementary Fig. 2a), Wnt agonist could stimulate epithelium formation in low temperature condition, which showed in Supplementary Fig. 2b, c. However, Wnt agonist was not able to proceed and complete the regeneration at low temperature (Supplementary Fig. 2d, e). These data indicated that Wnt signal is necessary but not sufficient for the successful tail regeneration.

### Low temperature increases activity of AMPKα during regeneration

**Fig. 6:**
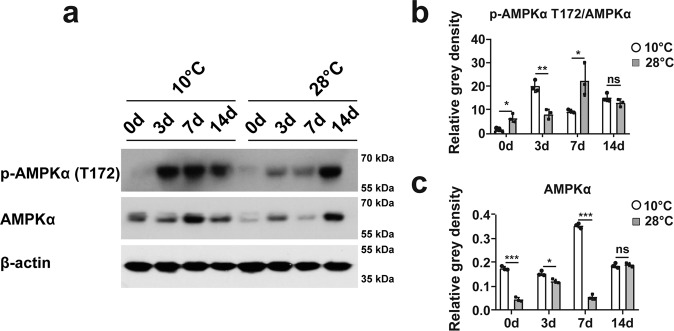
Low temperature increased activity of AMPKα during regeneration. **a** The variations of AMPKα and p-AMPKα (Thr172) in the high and low temperature groups on days 0, 3, 7, and 14. **b**, **c** Relative level of p-AMPKα (Thr172) and AMPKα in Fig. 6a. Values represent mean ± SD, *N* = 3, ^ns^*p* > 0.05, *p < 0.05, **p < 0.01, ***p < 0.001.

AMPKα is an important energy-sensing factor that monitors variations in ATP concentration. Studies have shown that this process occurs through regulation of mTOR. Samples of the newly grown tail tissues were collected and analyzed by western blotting. The results revealed that the level of phosphorylated AMPKα (Thr172) was significantly higher in the geckos in the 10 °C group compared to that in the 28 °C group on days 3 and 7 (Fig. 6a, b). Moreover, the expression of total AMPKα in 10 °C group was higher than that in the 28 °C group on day 0, 3 and 7. The expression of AMPKα reached its peak on day 7 in the 10 °C group, and then declined over time (Fig. 6a, c). These data suggested that the higher activity of AMPK at early stage might suppress the initiation of regeneration.

### mTOR inhibition and AMPK activation reduce the proliferation of wound epithelium

**Fig. 7:**
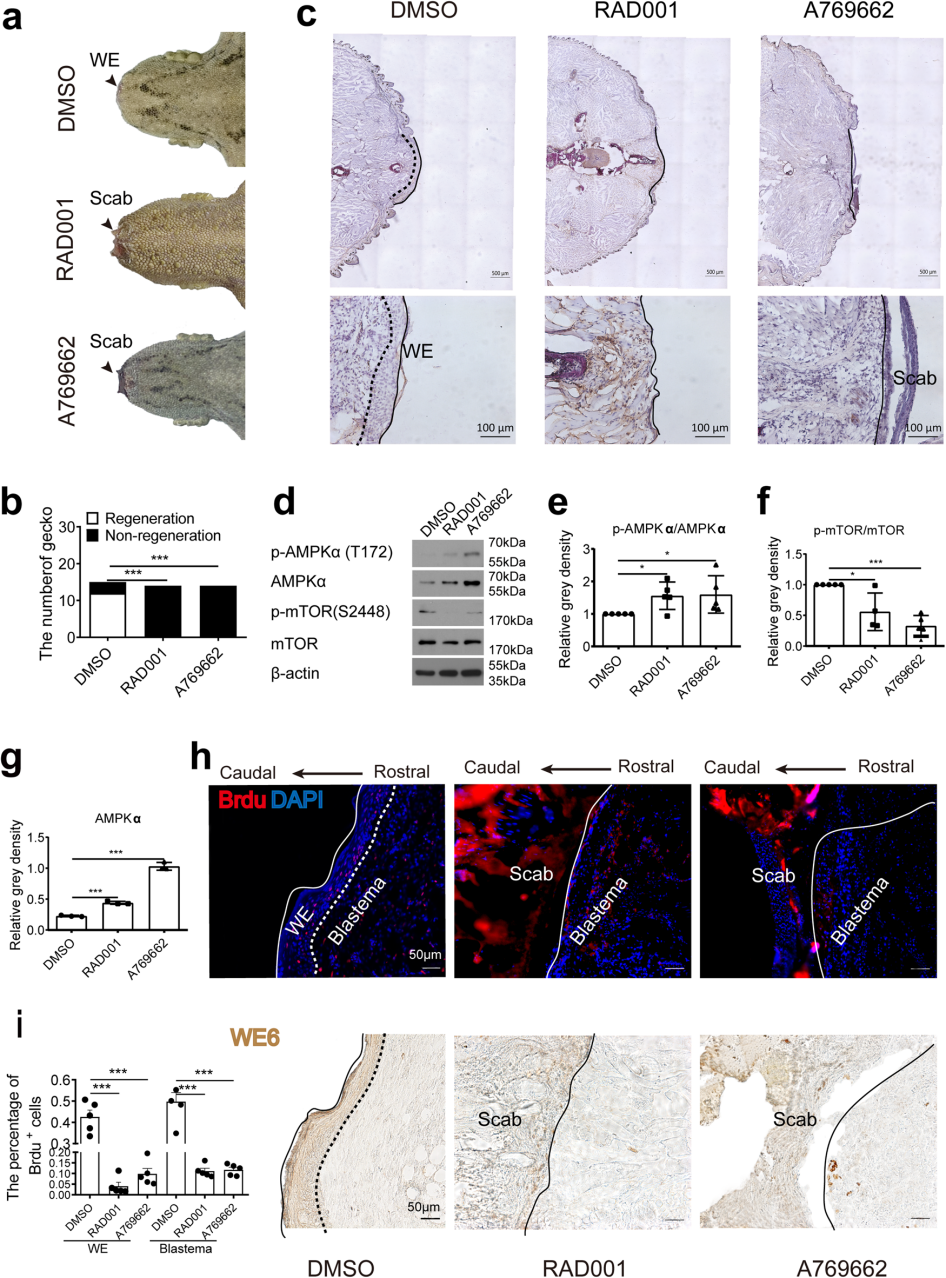
AMPK activation reduced the proliferation of wound epithelium. **a** Formation of wound epithelium (WE) in control (DMSO), mTOR inhibition (RAD001), and AMPK activation groups (A769662). **b** Number of geckos with regeneration in control, RAD001, and A769662 groups. *N* = 15. **c** H&E staining of tail tissues in control, RAD001, and A769662 groups. Solid arrow indicates the injury site, dashed box indicates the magnified area, dotted line indicates border between wound epithelium and scab, and solid line indicates junction between rostral end of wound epithelium and blastema. Scale bar = 500 µm or 100 µm. *N* = 3. **d** Protein level of AMPKα, p-AMPKα (Thr172), mTOR, and p-mTOR (Ser2448) in tail tissues. **e**–**g** Relative level of proteins in Fig. 7d. **h** Immunofluorescence analysis of BrdU staining in control, RAD001 and A769662 group (upper panel). Immunohistochemical analysis of WE6 staining in the same location of the samples. Solid line indicates the caudal border of wound epithelium, dotted line indicates border between wound epithelium and blastema. Scale bar = 50 µm. **i** Number of BrdU positive cells in control, mTOR inhibition, and AMPK activation groups. Values represent mean ± SD, *N* = 5, ^ns^*p* > 0.05, *p < 0.05, **p < 0.01, ***p < 0.001.

To determine the necessity of mTOR activation for tail regeneration in geckos, RAD001, a small molecule inhibited the activation of mTOR, was administered by intraperitoneal injection. Similarly, the ATP sensing protein AMPK, a negative regulator of mTOR, was activated by the intraperitoneal injection of agonist A769662. Wound epithelium formation was analyzed on 7 days post-surgery. The results showed that the formation of wound epithelium was significantly blocked in both the mTOR inhibition and AMPKα activation groups (Fig. 7a, b). No individuals with regenerated tails were found in the mTOR inhibition or AMPKα activation groups (Fig. 7b). In addition, the results were further validated by morphological assay that also demonstrated the occurrence of only scar tissues in both the mTOR inhibition (RAD001) and AMPKα activation groups (A769662). Intact wound epithelium tissues were observed in the DMSO group (Fig. 7c). To confirm the effects of the inhibitor and agonist, Western Blotting analysis was performed to determine the expression of mTOR and AMPK. The results showed that the ratios of p-AMPKα/AMPKα were significantly increased in the RAD001- (Fig. 7d, e) and A769662-treated groups (Fig. 7d, e). Total AMPKα was also increased in the RAD001- and A769662-treated groups (Fig. 7d, g). Phosphorylated mTOR/mTOR was significantly decreased in the RAD001-treated group (Fig. 7d, f) and the A769662-treated group (Fig. 7d, f). To investigate cell proliferation in the wound epithelium, BrdU incorporation was analyzed in the geckos. The results showed that the number of BrdU positive cells was significantly decreased in RAD001- and A769662-treated group (Fig. 7h, upper panel, Fig. 7i). WE6 was used to shape the site of wound epithelium (Fig. 7h, lower panel). To confirm the effect of mTOR inhibition on the formation of wound epithelium, another mTOR inhibitor, Torin1, was administrated as well, the treatment obviously inhibited tail regeneration (Supplementary Fig. 3a, b). The treatment also blunted the level of phosphorylated mTOR (Supplementary Fig. 3c, d). The BrdU positive cells was significantly decreased in Torin1-treated group (Supplementary Fig. 3f, upper panel, Fig. 3e). WE6 was used to shape the site of wound epithelium (Supplementary Fig. 3f, lower panel). Taken together, inhibition of mTOR and activation of AMPK may hinder the proliferation of wound epithelium.

## Discussion

Energy supplements maintain cellular activities and influence cell cycle and growth^9^. Energy metabolism is regulated by many signaling pathways and affected by the surrounding temperature, especially in the heterothermic animals^6^. Thus, heterothermic animals could be applied as a model to investigate the biological process that involved in metabolic regulation. In this study, we focused on the role of Akt/mTOR activation in energy deficiency situations during the tail regeneration of geckos. Our results revealed that the injury induced Wnt signal and the energy sensing AMPK pathway coordinately modulated the activity of mTOR and affected epithelium proliferation.

As an upstream signaling molecule, Wnt is strongly implicated in cell proliferation, differentiation, and regeneration process in response to injury^32–34^. Our results revealed that Wnt pathway promoted wound epithelium formation and Wnt inhibition significantly decreased Akt activation. Furthermore, we addressed whether Wnt signal regulating Akt activity. Previous studies for the cross-talk of Wnt and Akt/mTOR pathway was described as inhibition of GSK3-β by Akt, which could lead to stable β-catenin and Wnt activation^35,36^. Our data revealed an opposite regulatory pathway from Wnt to Akt, and this effect might be mediated by other growth factors indirectly. Therefore, we proposed a possible link between injury induced Wnt signal and metabolic related pathway. It might be essential for the appendage regeneration when a robust proliferation is required to initiate and sustain the regeneration process.

AMPK, an upstream regulator of mTOR, plays an important role in regulating the energy balance in cells during the regeneration process. Several studies have suggested that the AMPK/mTOR axis maintains the energy status and thus coordinates cellular activities^9,15,32^. To confront harsh winter conditions and food deficiency, geckos survive by hibernating and thus minimizing their energy expenditure. How can geckos maintain a lower metabolic level, a potential approach is to sustain a high activity of AMPK when confronting low temperature. In this study, we found that the expression of total AMPK was higher at low temperature than that at high temperature at the early stages of regeneration in geckos. Moreover, the robust expression of phosphorylated AMPK following surgery provided evidence for the retardation of wound epithelium at low temperature in geckos. Then another interesting question is that how the activity of AMPK is maintained or even increased in low temperature. Usually, as an enzyme, the activity is positively correlated with the temperature. How the structure of AMPK is orchestrated upon low temperature is an amazing topic in future study.

**Fig. 8:**
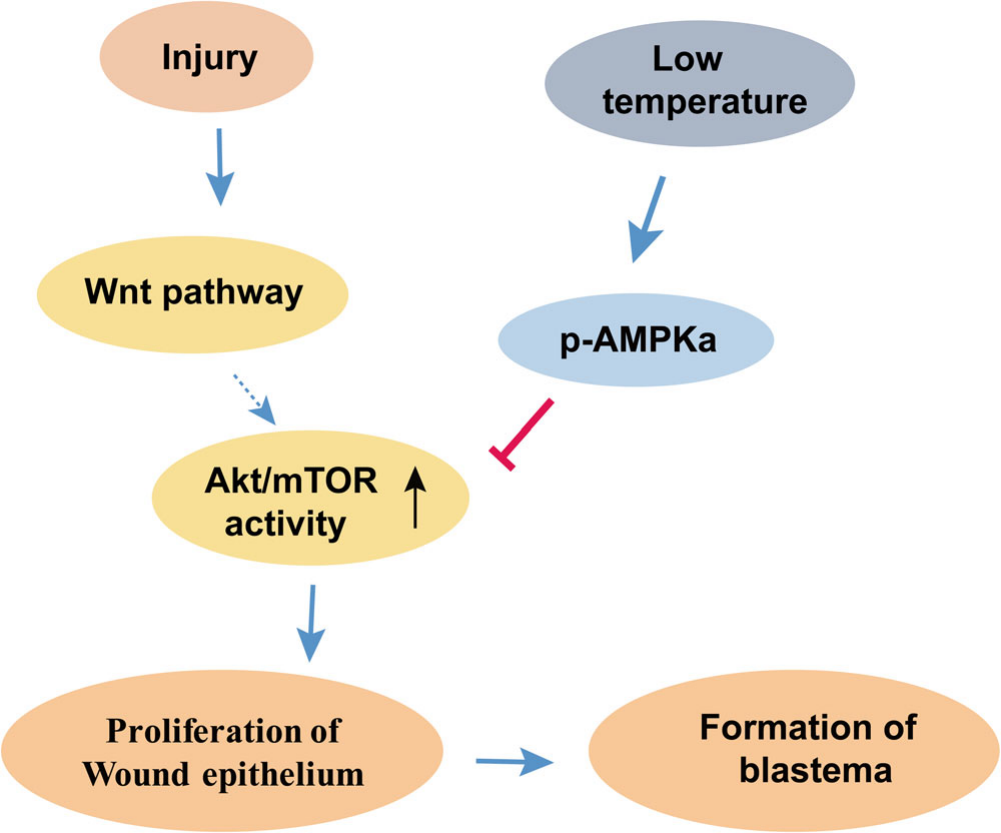
Schematic representation of working model. Schematic illustration of the Wnt pathway and low temperature regulated formation of wound epithelium and blastema. In response to tail shedding in the geckos, Wnt pathway is activated and then enhances the activity of Akt/mTOR. In condition of low temperature, the metabolic level of gecko decreases thus activates the AMPK activity, which in turn restrains the activity of mTOR. The increased Akt/mTOR activity is necessary for the proliferation of wound epithelium and formation of blastema.

In summary, as illustrated in Fig. 8, we demonstrated that low temperature and AMPK activation led to a downregulation of mTOR activity. The Wnt pathway is evoked by the injury signal and sequentially boosts the Akt activity. The AMPK and Wnt pathway exhibitesd opposing effects on the activity of mTOR, which is involved in regulating proliferation of the wound epithelium.

## Methods

### *Gekko japonicus* breeding and modeling

Adult *G. japonicus* with intact tails were grouped into control and experimental groups after health evaluation by veterinarians. The number of males and females were equal in each group. The average body weight was 4.4 ± 0.495 g. All geckos had free access to mealworms and sterilized water during the entire experiment. The culture incubator was set at 28 °C, 17 °C or 10 °C based on the test protocol. All animal experimental protocols were approved by the Institutional Animal Care and Use Committee of the Nantong University (Anima protocol: S20190405-402).

Tail autotomy was exerted following the protocols issued previously by our lab members^24^. Concisely, caudectomy was performed by locating a nylon slipknot at the 6th tail segment and then pulling gently to simulate the stimulation of autotomy in the natural environment. The successful regeneration was determined by the featured viewing as scab shedding and exposing of pink epithelium at ~7 day. The delayed scab shedding indicated deficiency of the wounding epithelium formation, which always led to failure of regeneration. The subsequent regeneration was evaluated by the length of regenerated tails that described in our previous study^24^, mainly detected by morphology observation, pathological investigation and molecular markers indication that detailed below.

### ATP extraction and evaluation

Liver and tail tissues near the 6th caudal vertebrae were collected and weighed, after which ATP lysis buffer (100 μL/10 mg sample, S0026, Beyotime Biotechnology, Nantong, China) was added into the tubes and the tissues were minced. The samples were incubated on ice for 5 min and then centrifuged at 12,000 × *g* at 4 °C. The supernatant (50 μL) was transferred into an opaque 96-well plate, which was pre-loaded with 100 μL ATP detection buffer. The absorbance of the samples was measured with an illuminometer (Synergy TM 2, BioTek, Waldbronn, Germany).

### Detection of metabolic parameters

The animals were maintained at either 28 °C or 10 °C for 1 week. Blood was drained from the hearts and the serum was separated by centrifugation at 12,000 × *g* for 10 min at 4 °C. The GLU, CHOL, TG, LDLC, HDLC, UA, and CR levels were determined using Siemens ADVIA® 2400 (Erlangen, Germany) based on the manufacturer’s instructions.

### Drug administration

The tails were amputated at the 6th caudal vertebrae to set up the regeneration models. Akt inhibitor MK-2206 (5 mg/kg body weight (BW) S1078, Selleck, Houston, TX) was administered after autotomy by i.p. injection every other day. BrdU solvent (20 μL of 20 mM solution, B5002, Sigma, Darmstadt, Germany) was administered on the last 2 days before sample collection. The samples were collected on the 7th day or other time point indicated. For the treatment of mTOR inhibitor (RAD001, Torin1) and AMPK activator (A769662, Selleck, Houston, TX), the gecko models were administered RAD001 (10 mg/kg BW, S1120, Selleck, Houston, TX), Torin1 (20 mg/kg BW, S2827, Selleck, Houston, TX) and A769662 (30 mg/kg BW, S2697, Selleck, Houston, TX) individually or together by i.p. injection post autotomy every other day. For the treatment of Wnt agonist, CHIR98014 was administered (5 mg/kg BW, S2745, Selleck, Houston, TX) by i.p. injection post autotomy every other day. The BrdU was administered as mentioned above. For treatment with the Wnt inhibitor, IWP-4 (2 mg/kg BW, SML1114, Sigma, Darmstadt, Germany) or XAV939 solution (40 mg/kg BW, S1180, Selleck, Houston, TX) was administered after amputation by i.p. injection every other day.

### Cell culture and drug treatment

Geckos with well-regenerated blastema at day 12 post autotomy were prepared. The anesthetized ones were sterilized with 70% EtOH and the blastema tissues were dissected out and digested in 0.25% trypsin-EDTA buffer at 30 °C for 10 min. Then cells were harvested and resuspended in 3 mL DMEM/F12 (10-092-CV, Thermo Fisher Scientific) containing 10% FBS, 2 mM glutamine (C0212, Beyotime, Nantong, China) and Penicillin-Streptomycin-Amphotericin B solution (100X, C0224, Beyotime, Nantong, China). Cells then were plated in 8-cm^2^ cell-culture dish under 30 °C and 5% CO2. The cells of passage 2 were gathered by conventional trypsin dislocation and allocated into a 6-well plate with 3 × 10^5^ cells per well for 12 h. The Wnt/β-catenin signaling agonist SKL2001 (40 μM, S8320, Selleck, Houston, TX) or CHIR98014 (8 μM, S2745, Selleck, Houston, TX) were applied into culture medium for additional 24 h before assays. The Akt antagonist MK-2206 (8 μM, S1078, Selleck, Houston, TX) were administrated as above.

### RNA isolation and quantitative real-time PCR

Cells were collected and total RNA was serparated by TRIzol (Vazyme, Nanjing, China). RNA pellets were qualified and quantified to be further reverse-transcribed with HiScript III 1st Strand cDNA Synthesis Kit (+gDNA wiper) (Vazyme, Nanjing, China), and qRT-PCR was performed using a TB Green® Premix Ex Taq™ II (Tli RNaseH Plus) (Takara, Beijing, China) following the supplier’s instructions. Primers were synthesized by Invitrogen company (Shanghai, China). Sequences of the primer were listed below: ef1α sense: 5'- GATGGAAAGTGACCCGCA-3', ef1α antisense: 5'-GAGGAAGACGCAGAGGTTTG-3', FGF7 sense: 5'-AGCCATGAACAAGTCTGGGA-3', FGF7 antisense: 5'-GCACCGTTCTGTTTCAATGC-3', FGF8 sense: 5'-AGAGCAACGGCAAAGG-3', FGF8 antisense: 5'-TGCTGGCGAGTCTTGG-3', FGF10 sense: 5'-GGCCACTAACTCTTCCTCGT-3', FGF10 antisense: 5'-CTTTCCCGTTCGGCTCAATC-3', FGF20 sense: 5'-TGGCAGTGGGTTTAGTCAG-3', FGF20 antisense: 5'-ACATTGGAGGAGTAAGTGTTGT-3'. All measurements were taken out at least in triplicates and data were normalized to endogenous ef1α expression. Gene expression was set as the fold change compared with the threshold cycle (Ct) and the relative expression levels were calculated by 2^-ΔΔCt^ method.

### Immunohistochemistry and immunofluorescence assay

The geckos were anesthetized by freezing on ice and perfused with pre-cooled 4% paraformaldehyde (PFA) solution. The blastema (5 mm before injury site) was sampled and subjected to 4% PFA fixation for 4 h and then washed with 0.1 M phosphate buffered saline (PBS) three times. The samples were then dehydrated in sequential concentrations of sucrose solution (5%, 10%, 20%, and 30%). The 12 μM sections were prepared from the OCT embedded blastema. For hematoxylin and eosin (H&E) staining, the sections were washed with PBS and double distilled water (DDW), stained with hematoxylin, eosin, and dehydrated by sequential concentration of ethanol solution. Sections were immersed in xylene three times and mounted with resin. Images were captured using an Olympus DP71 microscope (Tokyo, Japan) for further analysis.

For BrdU immunofluorescence detection, sections were warmed at 37 °C and rinsed with PBS. Sections were soaked in 1.5 M HCl for 30 min in a 37 °C water bath. After neutralization in 0.02 mM sodium tetraborate solution for 10 min at room temperature (RT) and PBS washes, the sections were then incubated with normal goat serum for 2 h at 37 °C. The extra blocking solution was drained and the sections incubated with anti-BrdU primary antibody overnight at 4 °C. The secondary antibody was incubated with the PBS-washed specimen for 2 h at RT. The sections were incubated with Hoechst 33342 antibody for 15 min at RT. Slides were mounted and observed by Zeiss M6 microscopy, and images were analyzed using Image Pro Plus. The fields of interested budding center were recorded and then merged for further BrdU positive cells counting. Data were collected from 5 gecko’s wound healing center area.

For immunofluorescence assay of cultured blastema cells, the samples were fixed with 4% paraformaldehyde for 20 min at 25 °C, then washed by PBS three times and blocked with 10 mg/mL bovine serum albumin, 0.1% Triton X-100 at 37 °C for 2 h. The cells were then incubated at 4 °C overnight with β-catenin antibody (1:200, Cat. ab32572, RRID: AB_725966, Abcam). After rewarmed at 25 °C for 30 min, cells were rinsed with PBS three times, and incubated with the corresponding Alexa Fluor 488-conjugated goat anti-rabbit IgG secondary antibody (1:400, Jackson ImmunoResearch, West Grove, PA, USA, Cat.111-545-003, RRID: AB_2338046) for 2 h at 25 °C. After counterstaining with DAPI (1:2500, Cat.D9542, Merck, Germany), the cells were washed with PBS and mounted to reduce photo bleaching. Fluorescence images were obtained using a Zeiss Axio Imager M2 microscope (Jena, Germany).

### Western blotting

Tail tissues (3 mm) before the injury site were excised and frozen in liquid nitrogen and stored at -80 °C. Protein samples were extracted and adjusted to identical concentration. Protein mixtures were loaded onto different percentage SDS-polyacrylamide gels (Bio-Rad) and transferred to polyvinylidene difluoride (PVDF) membranes (Millipore). Primary antibodies used were as follows: Akt (1:1000, 4685, Cell Signaling Technology (CST)), p-Akt Ser473 (1:1000, 4060, CST), p-Akt Thr308 (1:1000, 13038 S, CST), AMPKα (1:1000, 5832, CST), p-AMPKα Thr172 (1:1000, 2535, CST), mTOR (1:1000, 2983, CST), p-mTOR (1:1000, 2976, CST, 2976), p-p70 S6K Thr389 (1:1000, 9205, CST), p70 S6K (1:1000, 2708, CST), LEF1 (1:1000, ab85052, Abcam), β-catenin (1:1000, ab32572, Abcam), WE6 (1:5, WE6-s, Developmental Studies Hybridoma Bank) and β-actin (1:2000, ab8826, Abcam). β-actin was used as an internal control.

### Statistics and reproducibility

All data are presented as mean ± SD values. Replicates are biologically representing independent experimental subject. Experiments were duplicated at least three times and sample size was qualified according to methodology and was specialized in figure legends. Two groups of data were compared using homogeneity and *t*-tests, and multiple groups of data were analyzed by One-way (one factor comparation) or Two-way (two factor comparation) ANOVA followed by Bonferroni post-hoc test using SPSS Statistics 22.0 (SPSS Inc., Chicago, IL, USA). Chi-square test was adopted in the analysis for regeneration rate. *p* < 0.05 was considered statistically significant.

### Reporting summary

Further information on research design is available in the Nature Research Reporting Summary linked to this article.

## Supplementary information

Supplementary Information

Description of Additional Supplementary Files

Supplementary data 1

Reporting Summary

## Data Availability

All data are contained within the manuscript and Supplementary Data files. The source data for graphs are provided in the Supplementary Data 1. Uncropped immunoblots are provided in the Supplementary Fig. 4.
