# Supplementary material for: Akt/mTOR integrate energy metabolism with Wnt signal to influence wound epithelium growth in Gekko Japonicus
Source: Commun Biol. 2022 Sep 27;5:1018. doi: 10.1038/s42003-022-04004-5 (PMC9515156; doi:10.1038/s42003-022-04004-5)
Supplement: Supplementary file 1 — Supplementary Information [file 42003_2022_4004_MOESM1_ESM.pdf]

Akt/mTOR integrate energy metabolism with Wnt signal to influence wound epithelium growth in *Gekko Japonicus*

Qinghua Wang, Zuming Mao, Zhuang Liu, Man Xu, Shuai Huang, Yin Wang, Yanran Xu, Longju Qi, Mei Liu, Yan Liu

Supplementary figures

Supplementary Figure 1 Wnt pathway promoted Akt activity and enhanced FGFs expression in blastema cells

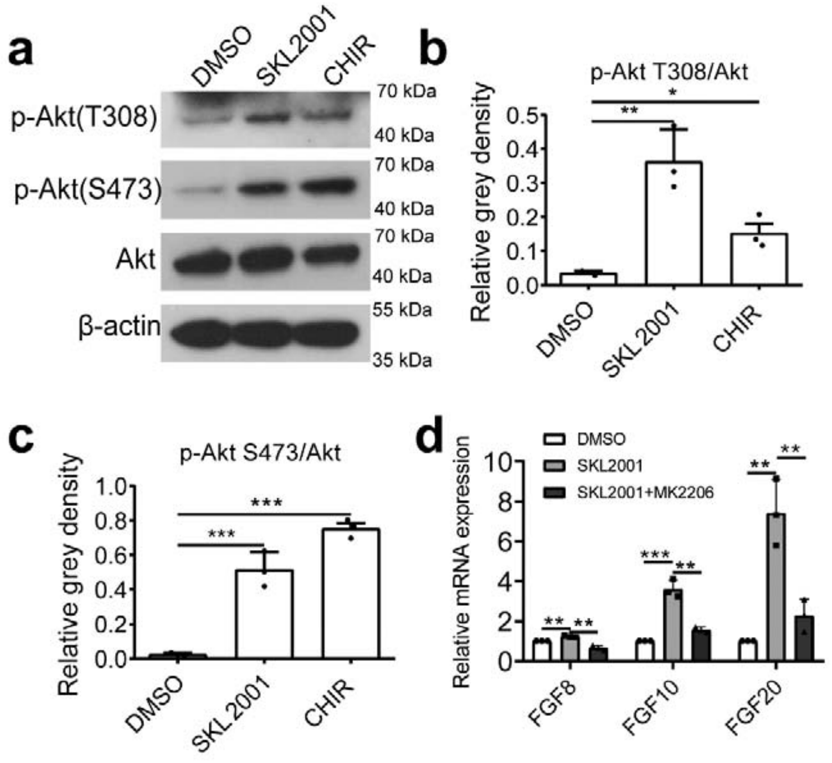

a, Wnt agonist (SKL2001 or CHIR) promoted Akt phosphorylation in cultured blastema cells. **b-c**, Relative level of p-Akt (Thr308)/Akt and p-Akt (Ser473)/Akt in **Supplementary Fig. 1a**. **d**, The transcriptional expression of FGF8, FGF10 and FGF20 were enhanced in SKL2001 treated blastema cells, while attenuated by administration of MK-2206 (Akt antagonist), N=3. Values represent mean ± SD, $^{ns}p > 0.05$, * $p < 0.05$, ** $p < 0.01$, *** $p < 0.001$.

Supplementary Figure 2 Wnt agonist partially rescued the deficit of wounding epithelium formation in low temperature during tail regeneration.

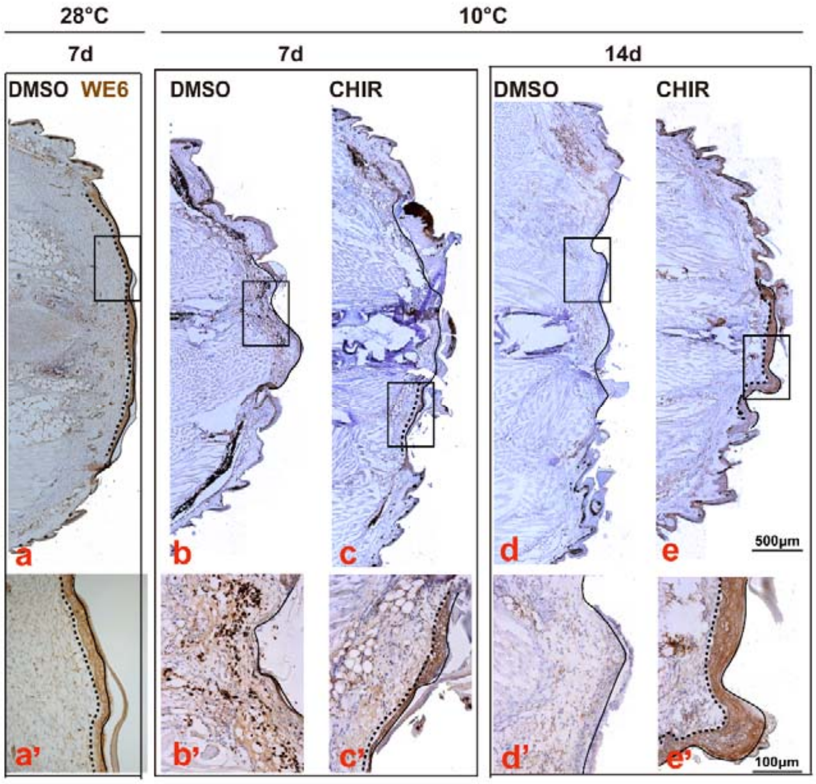

a, Wounding epithelium positively stained with WE6 antibody were observed at day 7 post tail amputation in normal temperature. **b-c**, The epithelium formation was inhibited in low temperature, while the treatment of Wnt agonist CHIR gave rise to partial formation of epithelium at day 7 post tail amputation in low temperature. **d-e**, The treatment of CHIR that produced partial epithelium could not proceed and complete regeneration after 14 days post amputation in low temperature condition. **a'-e'** were the enlarged views of boxed areas in **a-e**, respectively. Solid line indicates the caudal border of wound epithelium, dotted line indicates border between wound epithelium and blastema.

Supplementary Fig. 3 mTOR inhibition by Torin1 hindered the regeneration wound epithelium

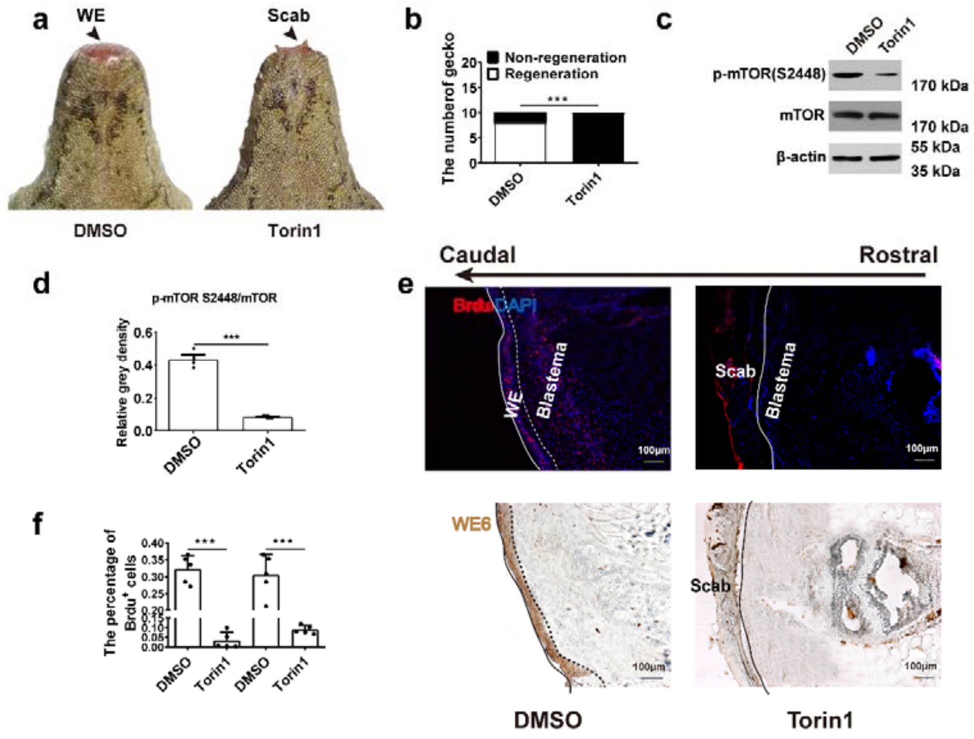

a, Formation of wound epithelium (WE) in the control (DMSO) and mTOR inhibition groups (Torin1). **b**, Number of geckos with regeneration in control and Torin1 groups. N=10. **c**, Protein level of p-mTOR (Ser2448), and mTOR in tail tissues. **d**, Relative level of p-mTOR (Ser2448)/mTOR. N=3. **e**, Immunofluorescence analysis of BrdU staining in DMSO and Torin1 group (upper panel). Immunohistochemical analysis of WE6 staining in the same location of the samples (lower panel). Scale bar = 100µm. Solid line indicates border between wound epithelium and scab, and dotted line indicates border between rostral end of wound epithelium and blastema. **f**, Number of BrdU positive cells in control and mTOR inhibition groups. N = 5. Values represent mean ± SD, ns $p > 0.05$, *$p < 0.05$, ** $p < 0.01$, *** $p < 0.001$.

Supplementary Fig. 4 Uncropped immunoblots

Raw immunoblotted films for Figure 2

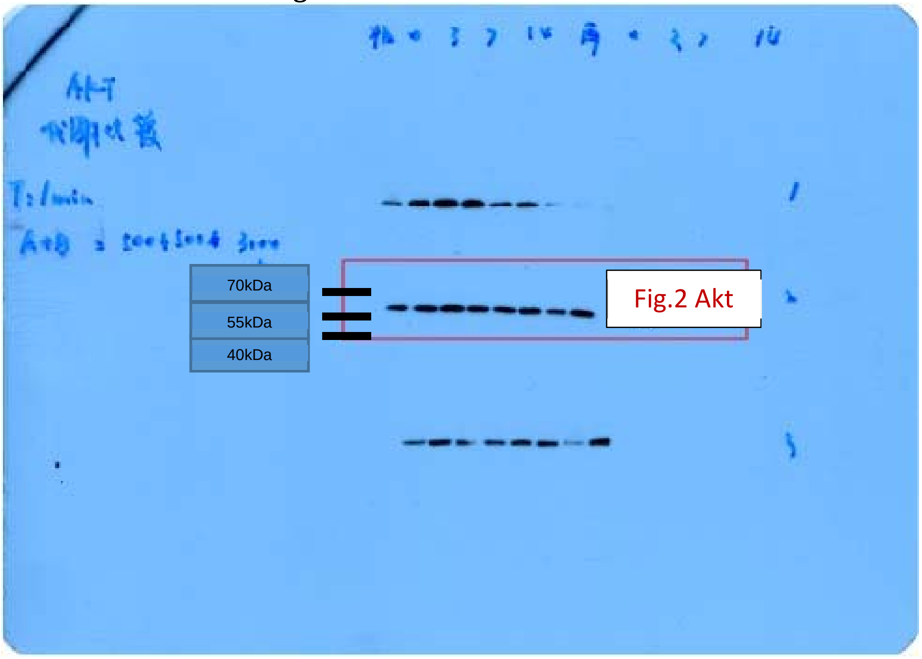

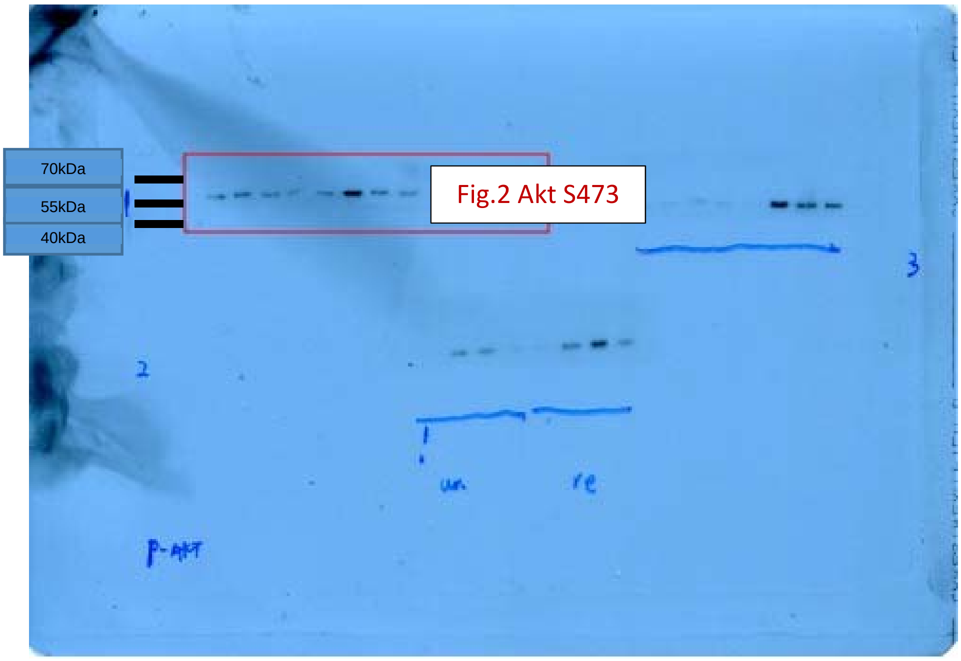

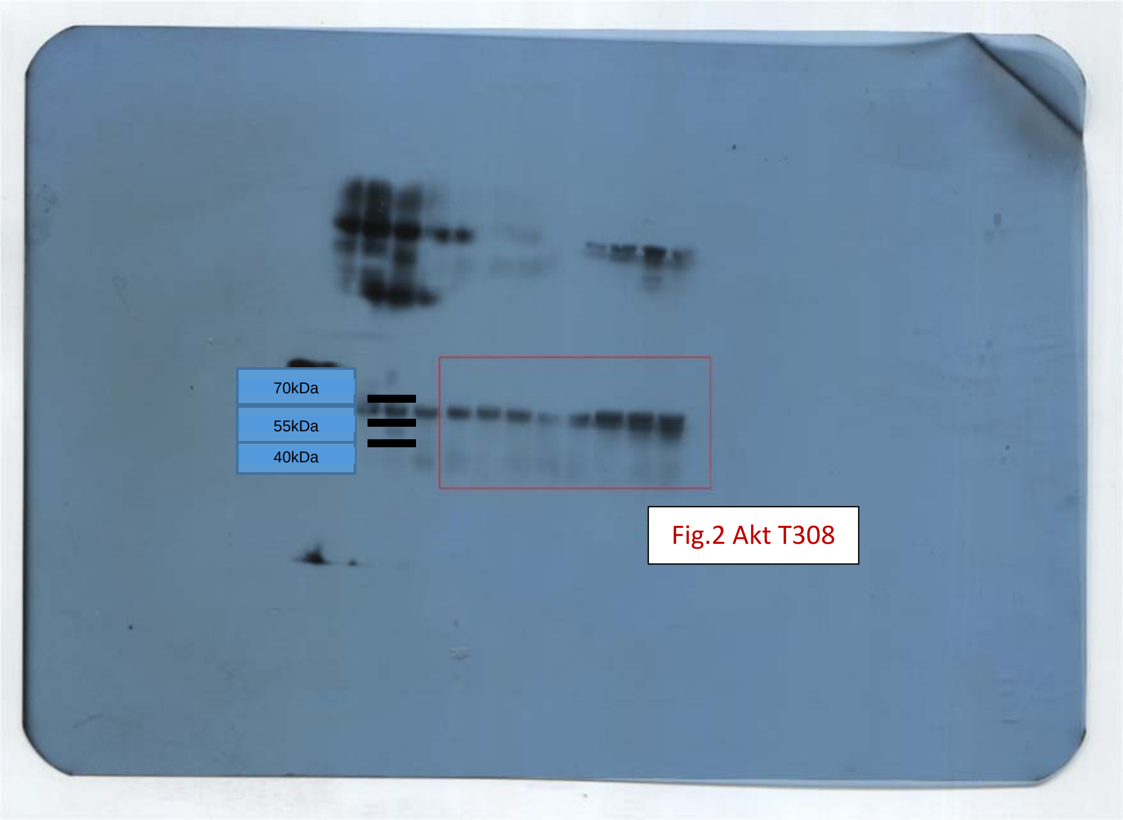
70kDa
55kDa
40kDa
Fig.2 Akt T308

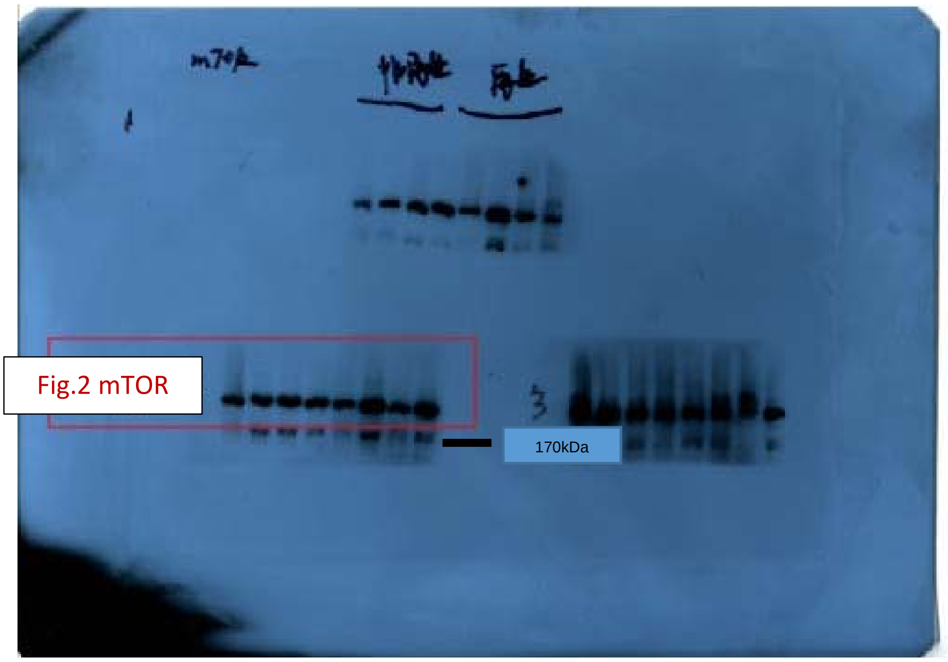
Fig.2 mTOR
170kDa

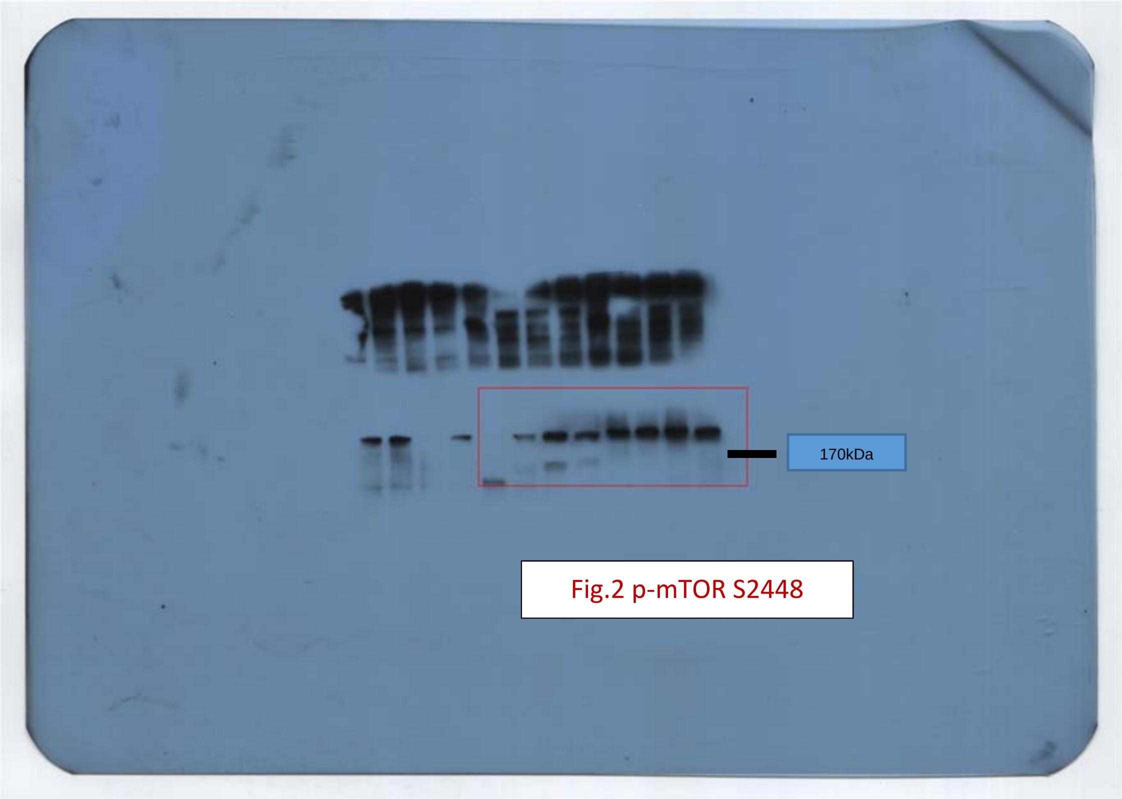

Fig.2 p-mTOR S2448

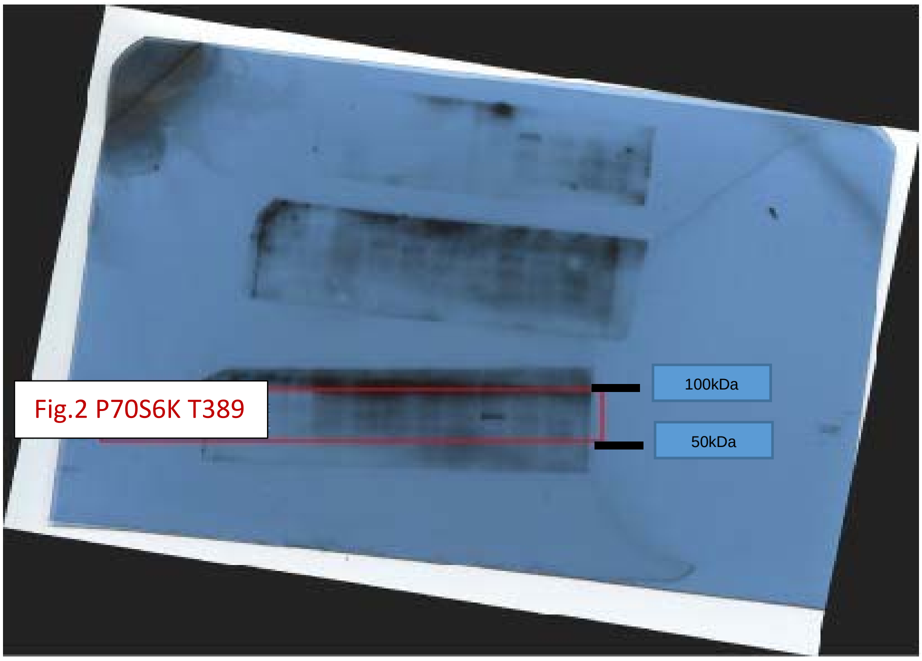

Fig.2 P70S6K T389

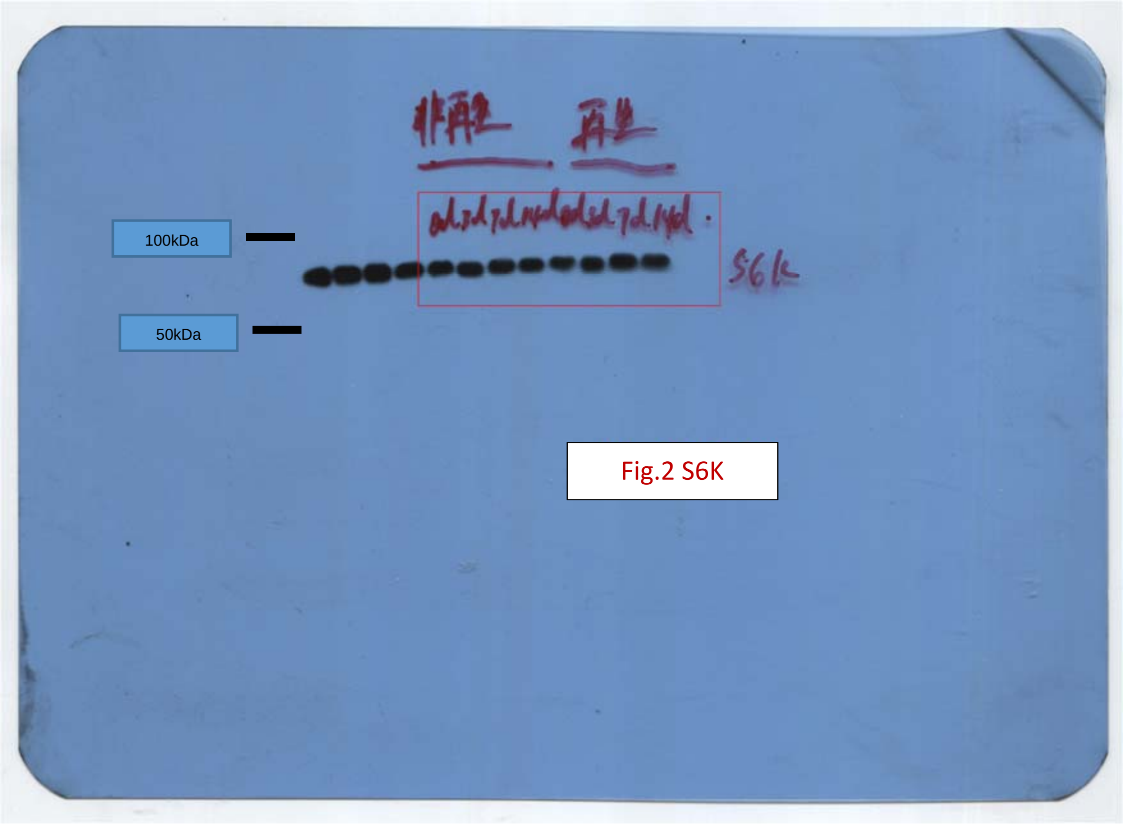

Fig.2 S6K

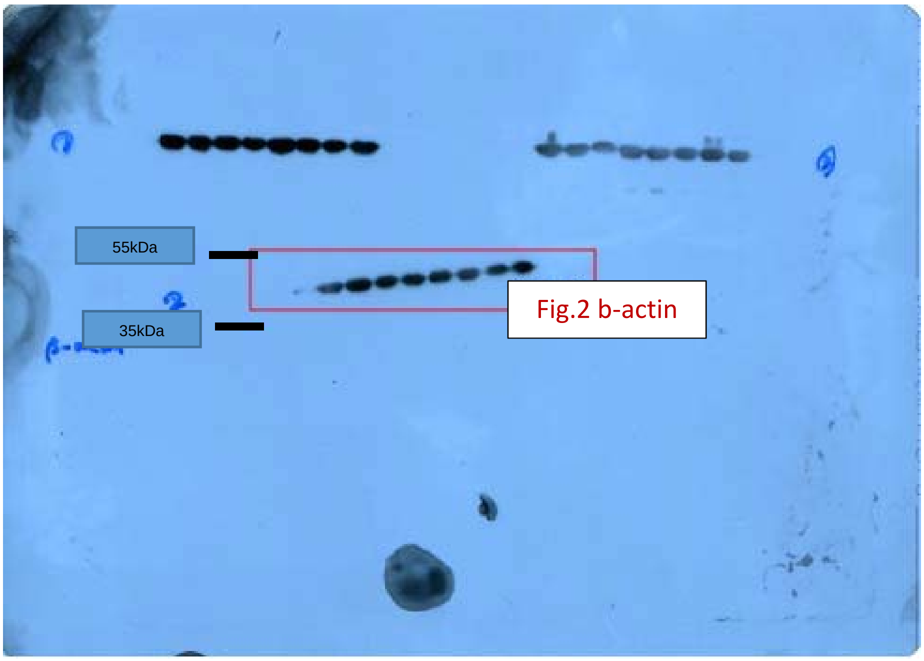

Fig.2 b-actin

Raw immunoblotted films for Figure 3

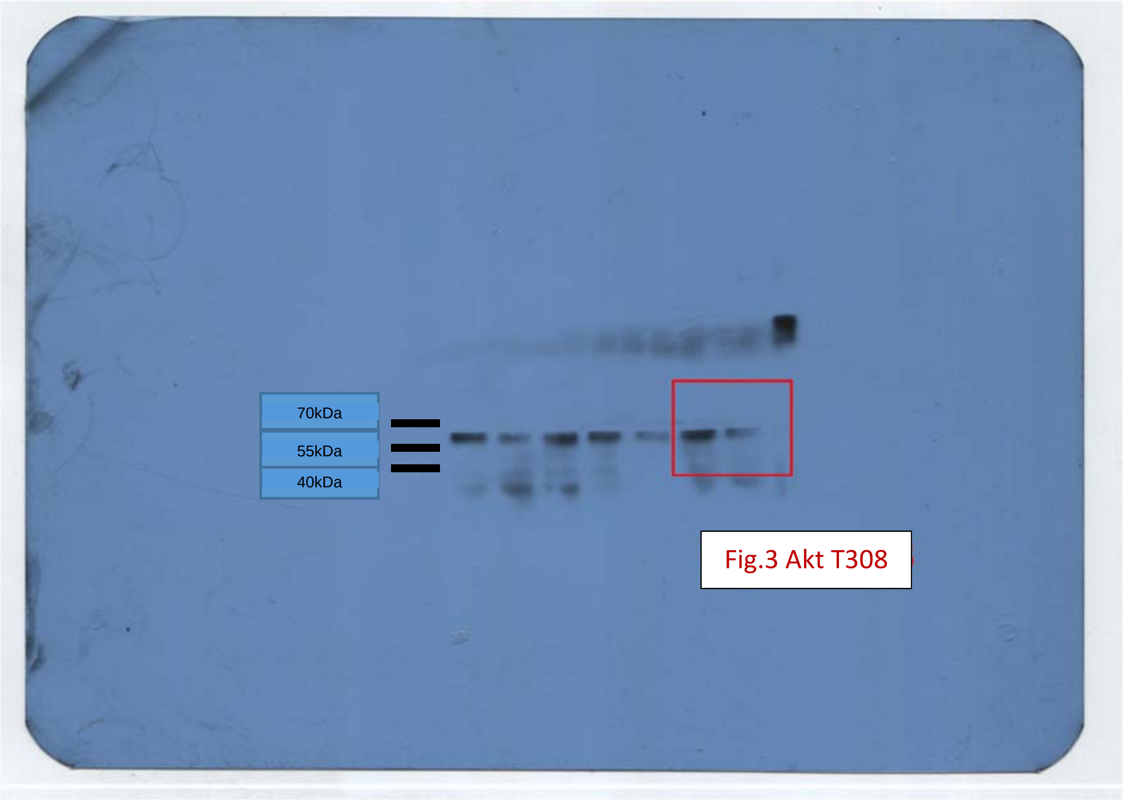

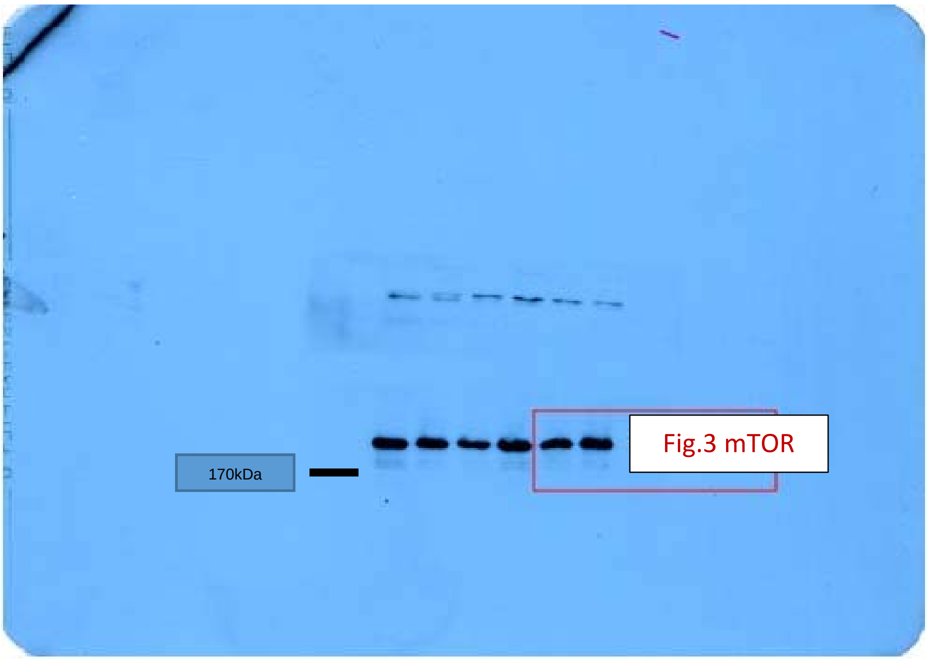

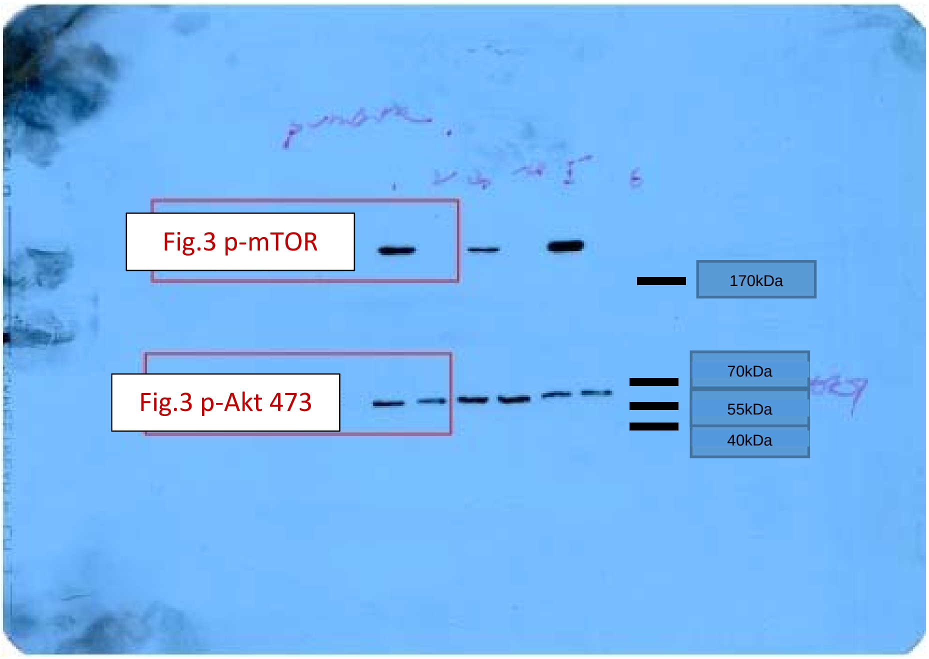
Fig.3 p-mTOR
170kDa
Fig.3 p-Akt 473
70kDa
55kDa
40kDa

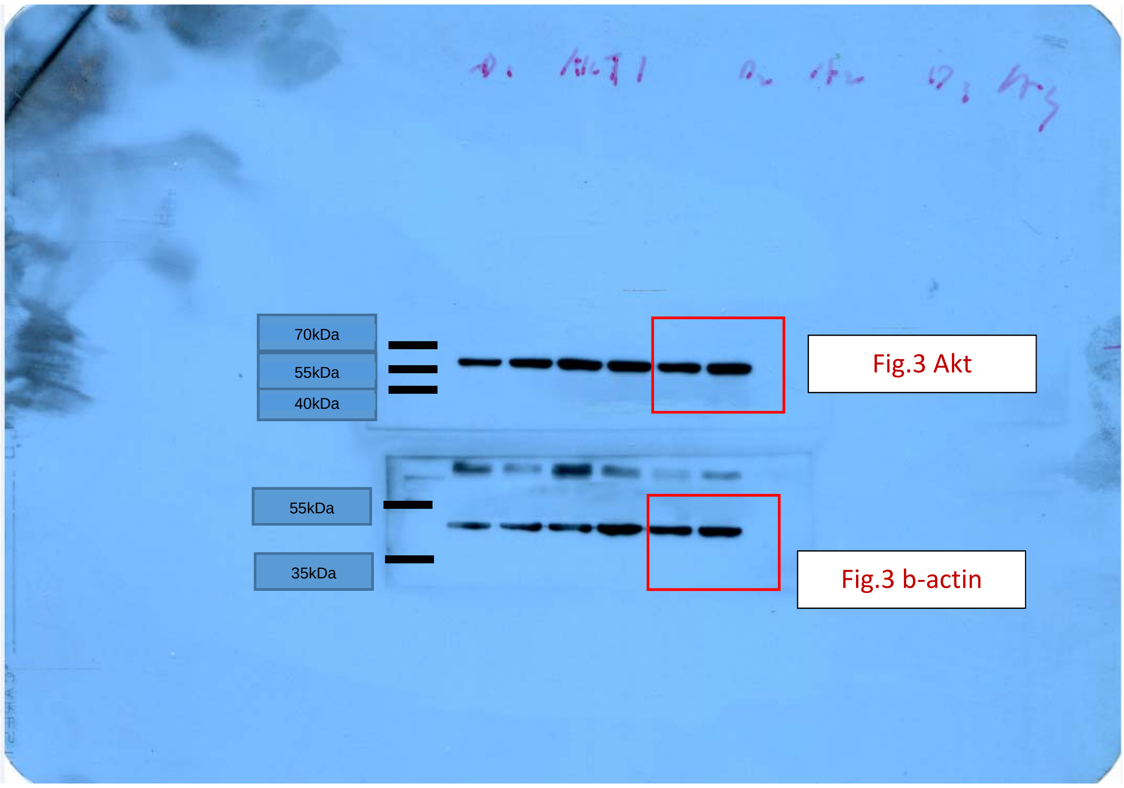
70kDa
55kDa
40kDa
Fig.3 Akt
55kDa
35kDa
Fig.3 b-actin

Raw immunoblotted films for Figure 5

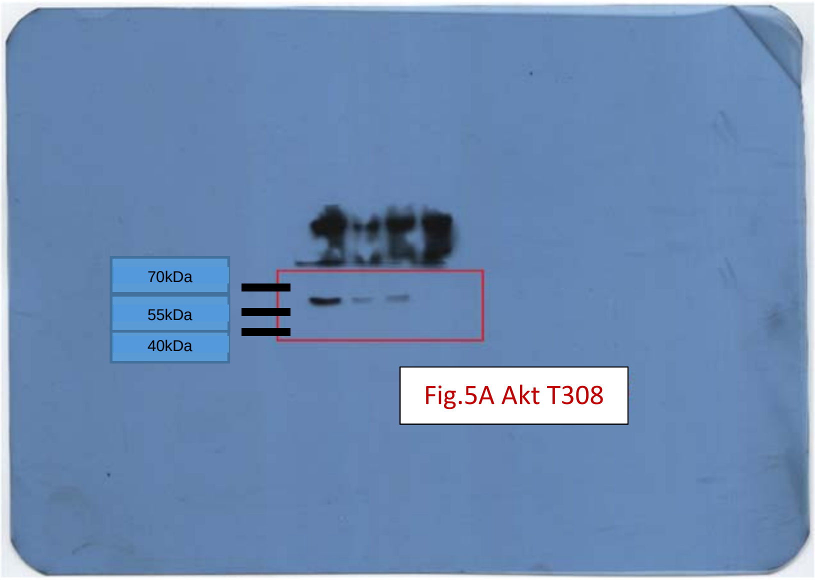

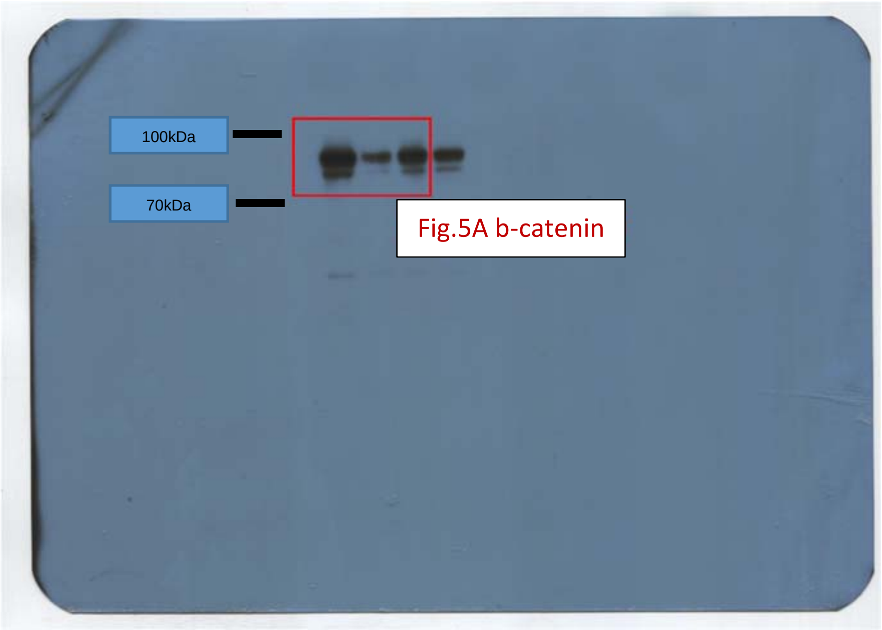

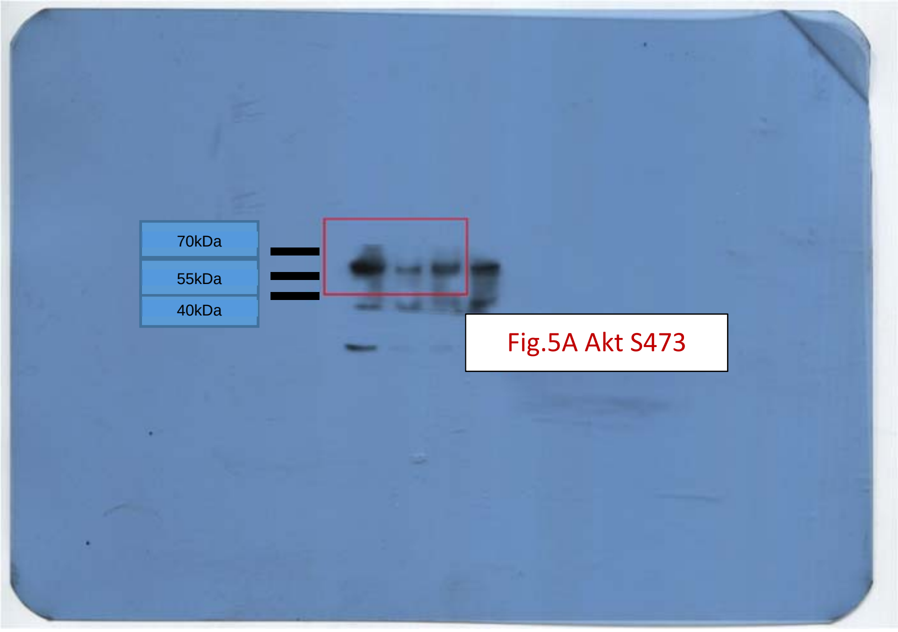
70kDa
55kDa
40kDa
Fig.5A Akt S473

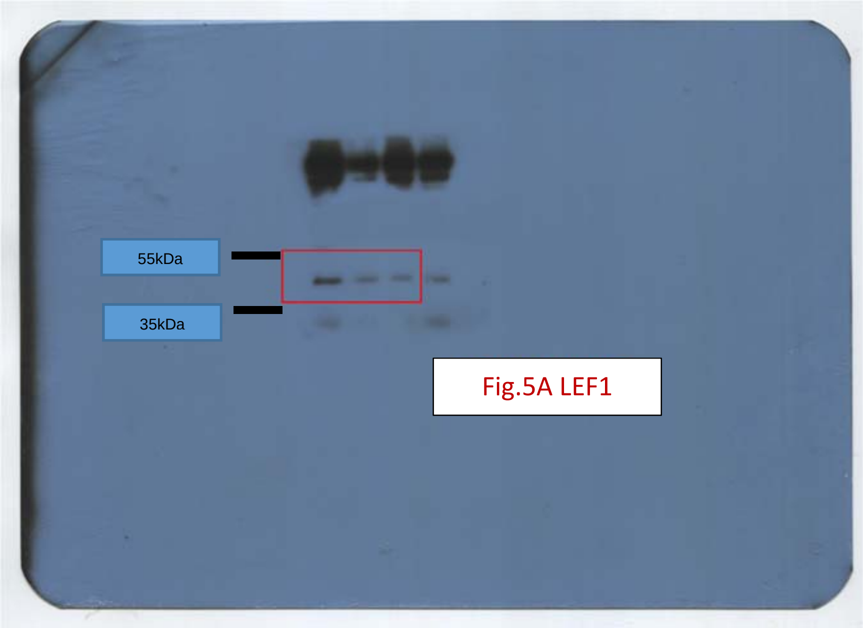
55kDa
35kDa
Fig.5A LEF1

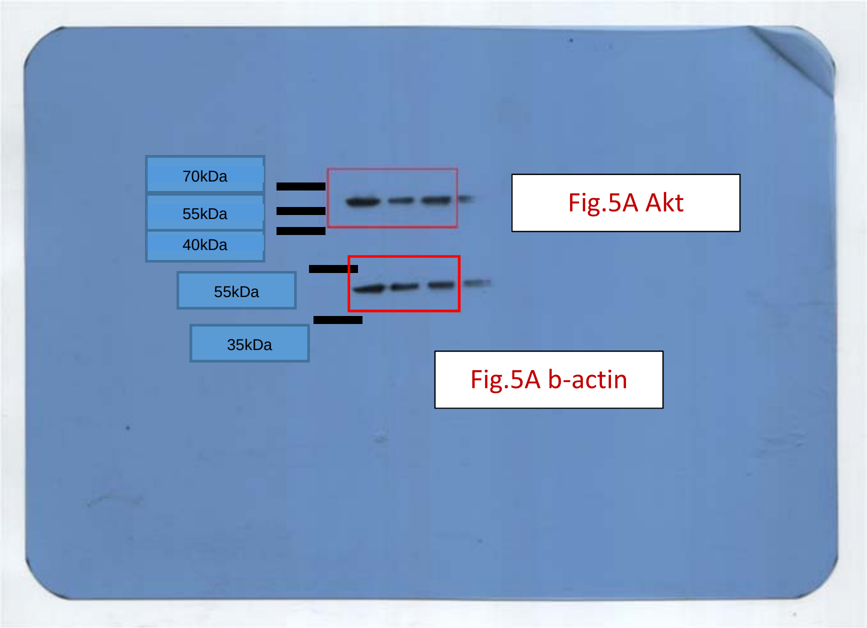
70kDa
55kDa
40kDa
Fig.5A Akt
55kDa
35kDa
Fig.5A b-actin

Raw immunoblotted films for Figure 6

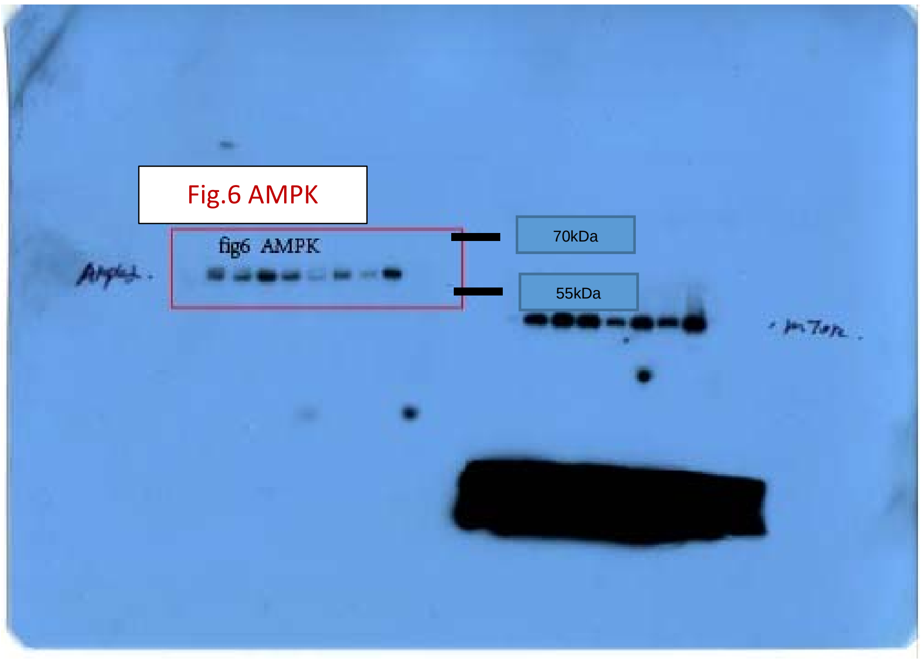

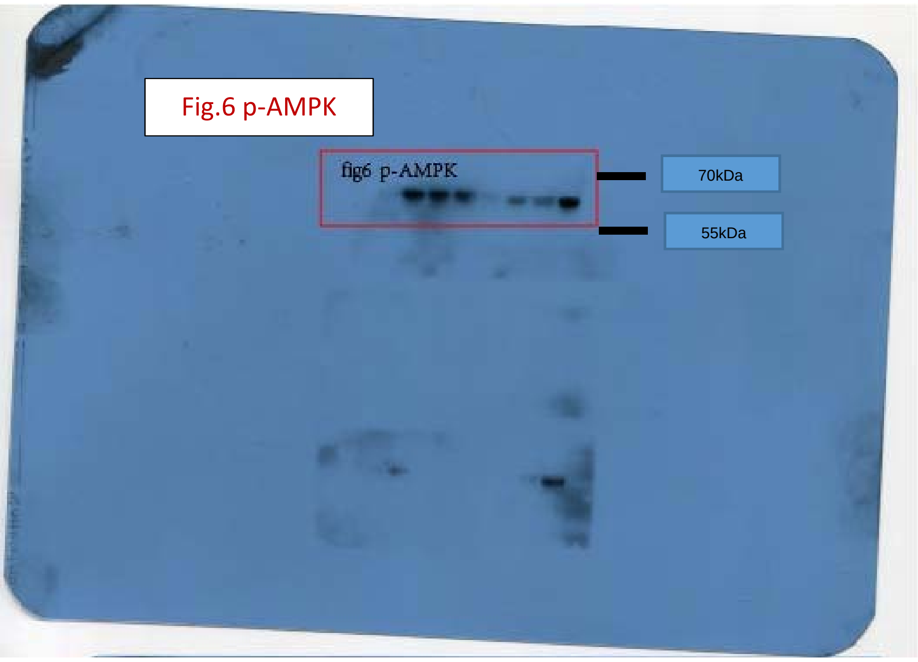

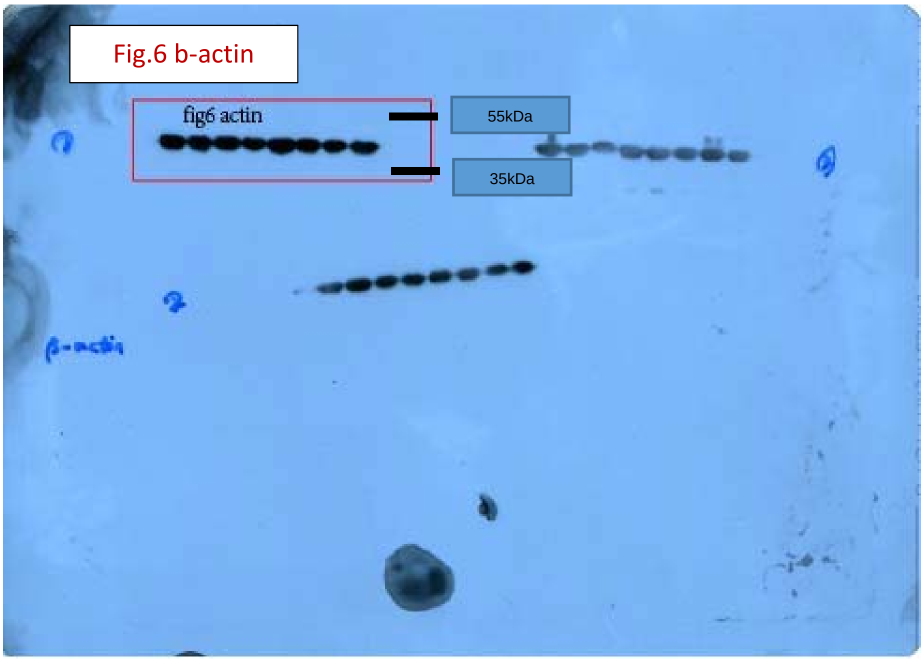
Fig.6 b-actin
fig6 actin
55kDa
35kDa
β-actin

Raw immunoblotted films for Figure 7

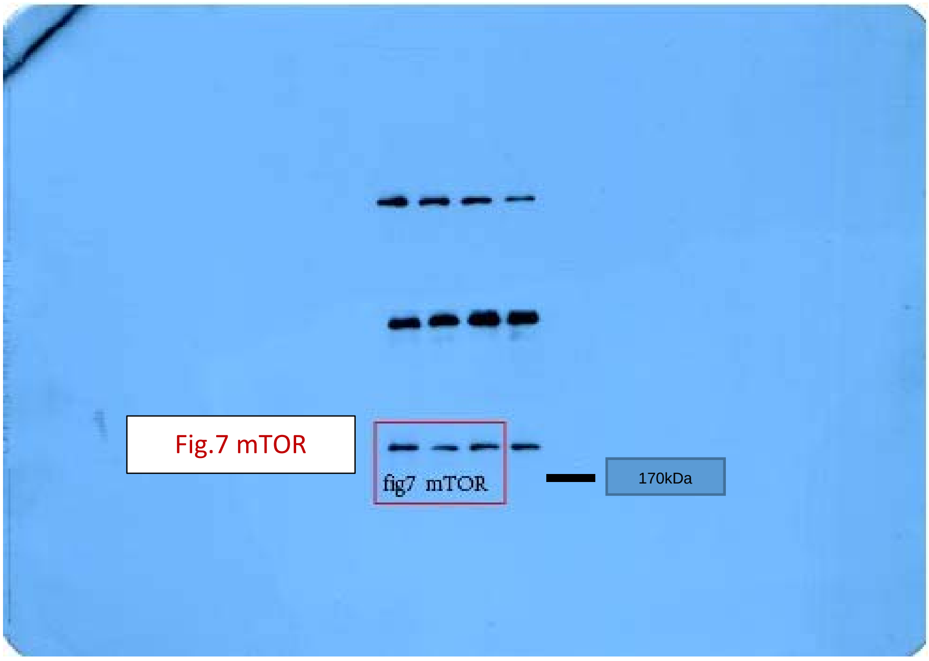

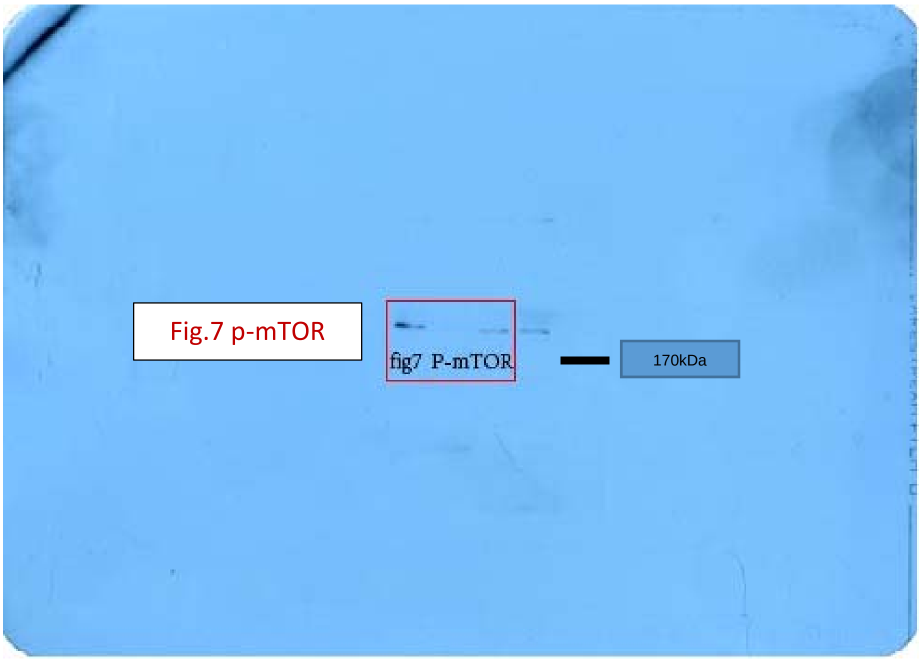

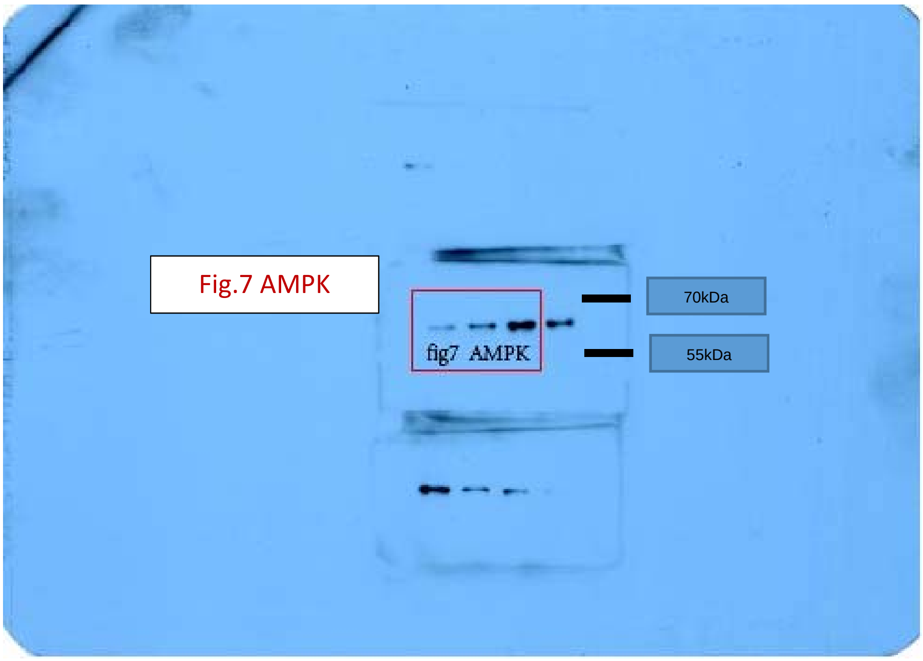
Fig.7 AMPK
fig7 AMPK
70kDa
55kDa

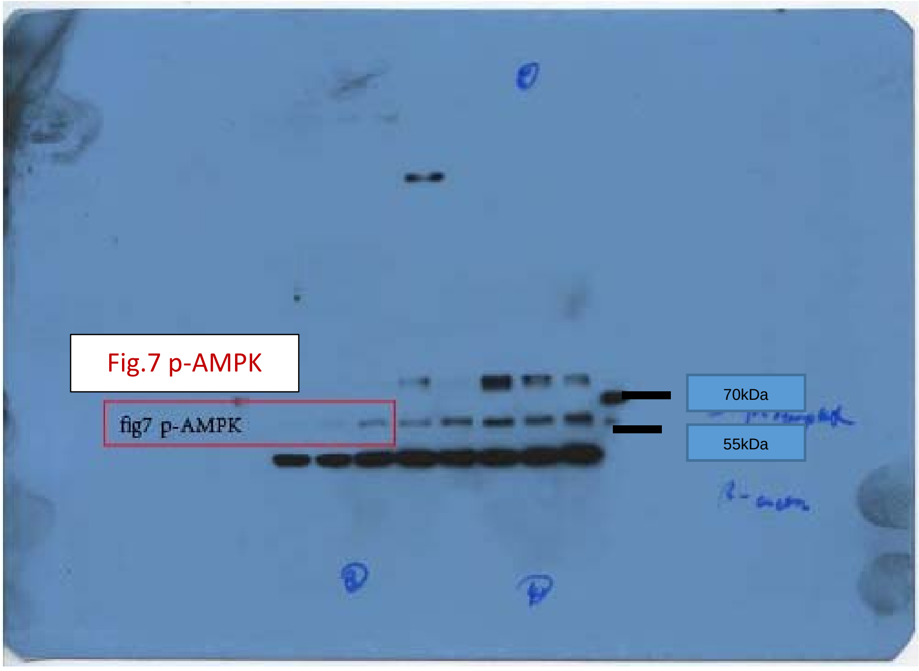
Fig.7 p-AMPK
fig7 p-AMPK
70kDa
55kDa

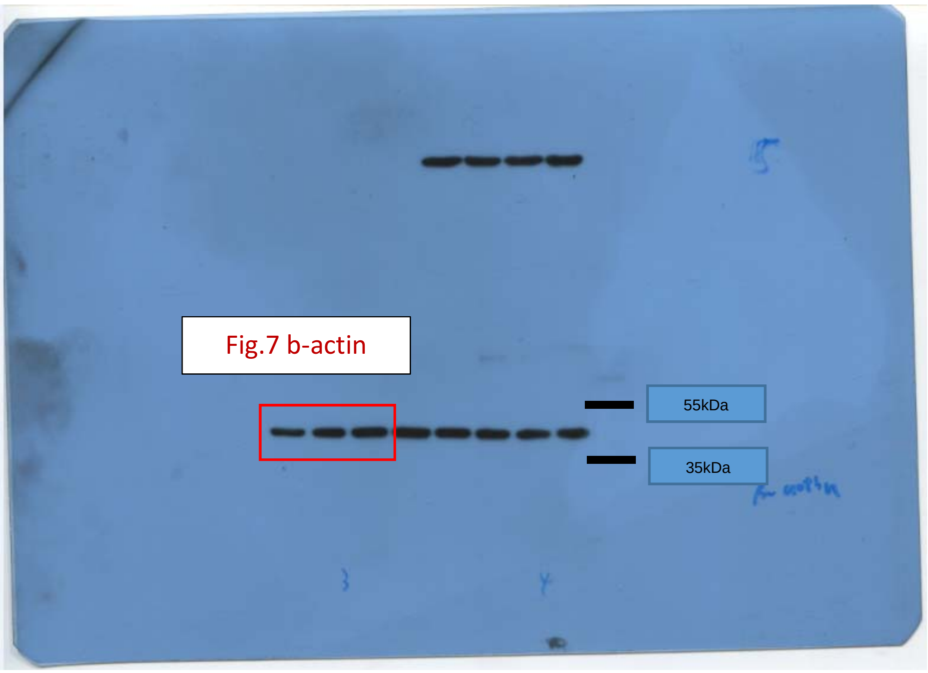
Fig.7 b-actin
55kDa
35kDa

Raw immunoblotted films for Supplementary Figure 1

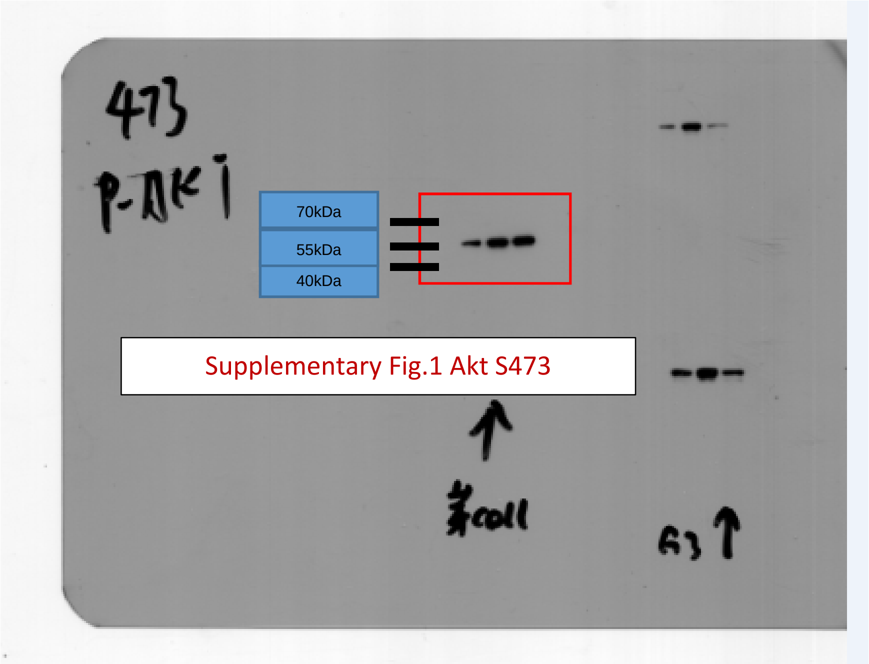

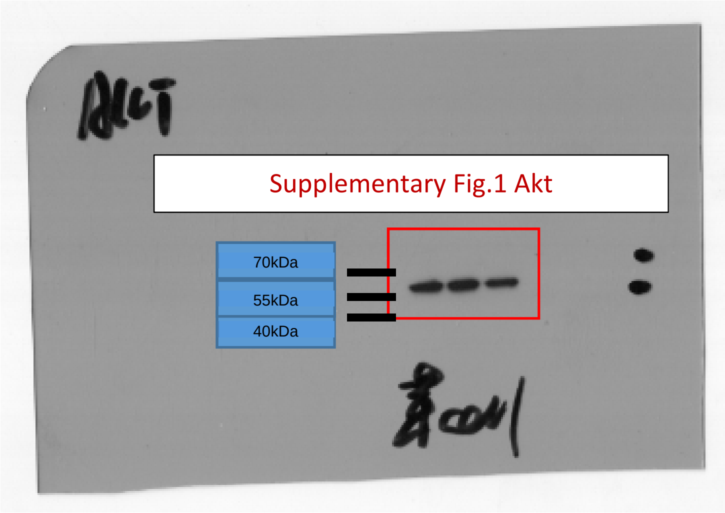

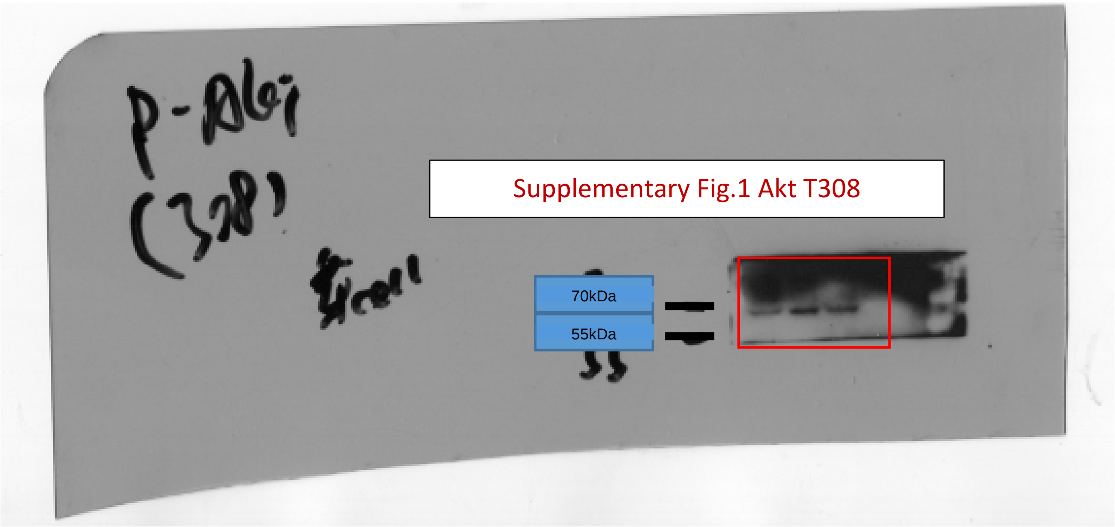
Supplementary Fig.1 Akt T308
70kDa
55kDa

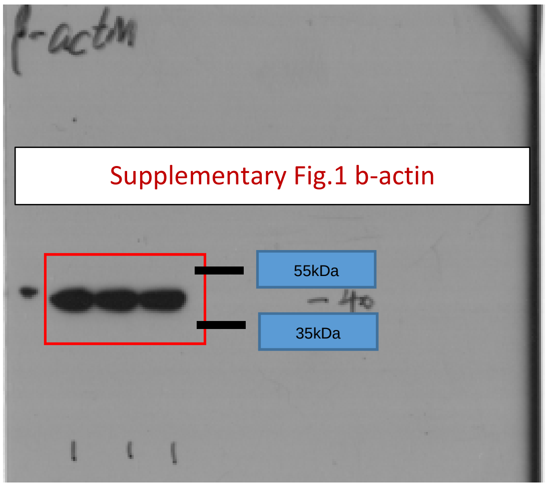
Supplementary Fig.1 b-actin
55kDa
35kDa

Raw immunoblotted films for Supplementary Figure 3

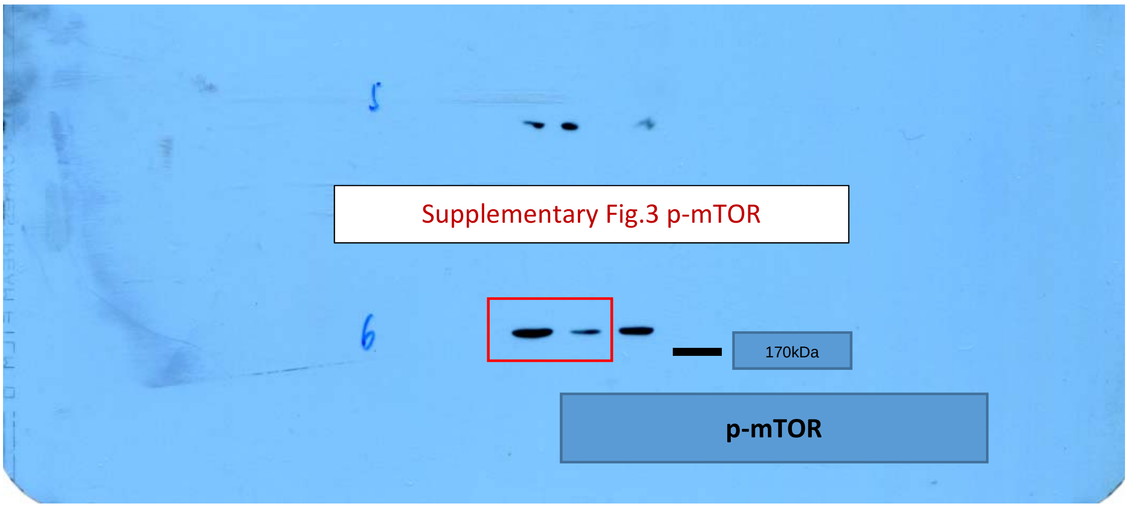

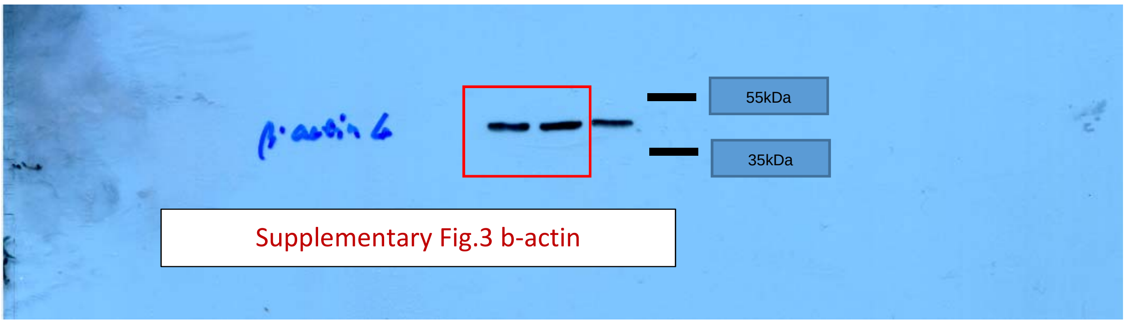

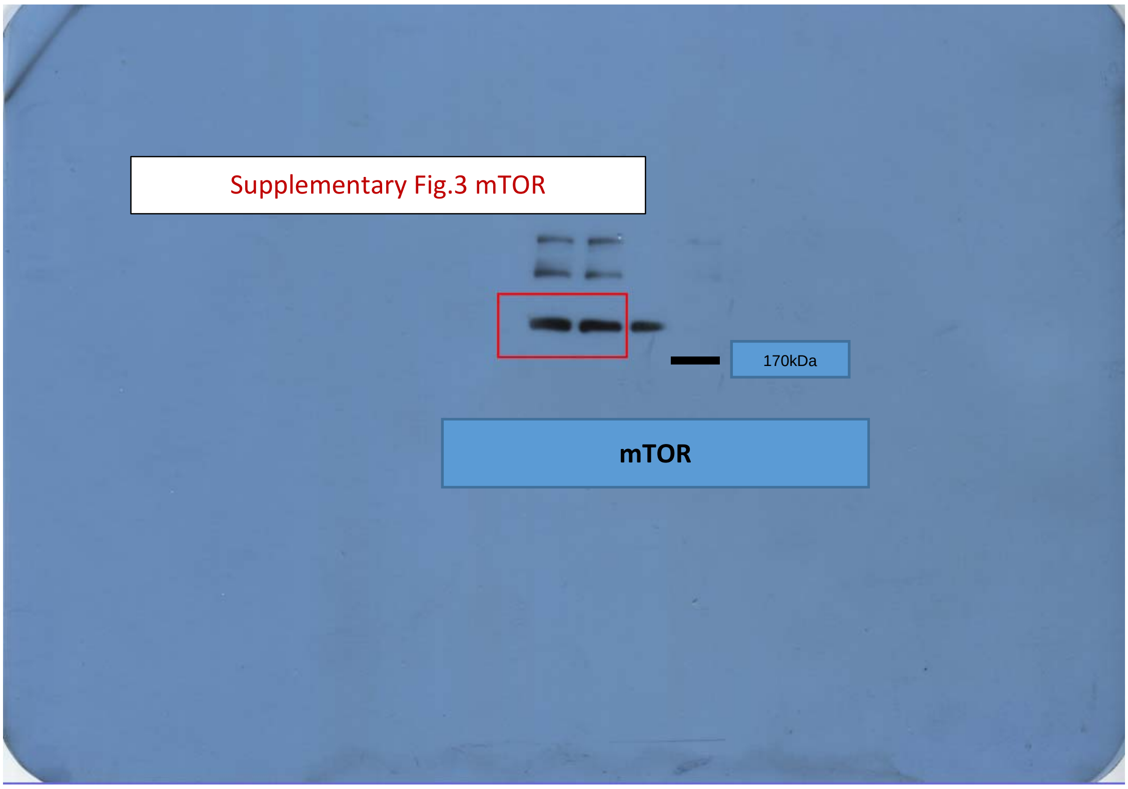
Supplementary Fig.3 mTOR
170kDa
mTOR
