# Supplementary material for: Akt/mTOR integrate energy metabolism with Wnt signal to influence wound epithelium growth in Gekko Japonicus
Source: Commun Biol. 2022 Sep 27;5:1018. doi: 10.1038/s42003-022-04004-5 (PMC9515156; doi:10.1038/s42003-022-04004-5)
Supplement: Supplementary file 4 — Reporting Summary [file 42003_2022_4004_MOESM4_ESM.pdf]

nature portfolio

Corresponding author(s): Yan Liu

Last updated by author(s): Sep 5, 2022

Reporting Summary

Nature Portfolio wishes to improve the reproducibility of the work that we publish. This form provides structure for consistency and transparency in reporting. For further information on Nature Portfolio policies, see our Editorial Policies and the Editorial Policy Checklist.

Statistics

For all statistical analyses, confirm that the following items are present in the figure legend, table legend, main text, or Methods section.

n/a	Confirmed	
☐	☒	The exact sample size (*n*) for each experimental group/condition, given as a discrete number and unit of measurement
☐	☒	A statement on whether measurements were taken from distinct samples or whether the same sample was measured repeatedly
☐	☒	The statistical test(s) used AND whether they are one- or two-sided<br>*Only common tests should be described solely by name; describe more complex techniques in the Methods section.*
☒	☐	A description of all covariates tested
☒	☐	A description of any assumptions or corrections, such as tests of normality and adjustment for multiple comparisons
☐	☒	A full description of the statistical parameters including central tendency (e.g. means) or other basic estimates (e.g. regression coefficient) AND variation (e.g. standard deviation) or associated estimates of uncertainty (e.g. confidence intervals)
☐	☒	For null hypothesis testing, the test statistic (e.g. *F*, *t*, *r*) with confidence intervals, effect sizes, degrees of freedom and *P* value noted<br>*Give P values as exact values whenever suitable.*
☒	☐	For Bayesian analysis, information on the choice of priors and Markov chain Monte Carlo settings
☒	☐	For hierarchical and complex designs, identification of the appropriate level for tests and full reporting of outcomes
☒	☐	Estimates of effect sizes (e.g. Cohen's *d*, Pearson's *r*), indicating how they were calculated

Our web collection on statistics for biologists contains articles on many of the points above.

Software and code

Policy information about availability of computer code

Data collection	Siemens ADVIA® 2400, DP2-BSW Ver.2.1 to Ver.2.2 (Build 6212), ImageJ software V 1.8.0 (NIH, NY, USA)
Data analysis	SPSS Statistics 22.0 (SPSS Inc., Chicago, IL,USA)

For manuscripts utilizing custom algorithms or software that are central to the research but not yet described in published literature, software must be made available to editors and reviewers. We strongly encourage code deposition in a community repository (e.g. GitHub). See the Nature Portfolio guidelines for submitting code & software for further information.

Data

Policy information about availability of data

All manuscripts must include a data availability statement. This statement should provide the following information, where applicable:

- Accession codes, unique identifiers, or web links for publicly available datasets
- A description of any restrictions on data availability
- For clinical datasets or third party data, please ensure that the statement adheres to our policy

All data are contained within the manuscript and Supplementary Data files. All uncropped immunoblots and original data that support the findings of this study can be found in Supplementary information and Supplementary data files, respectively

Human research participants

Policy information about studies involving human research participants and Sex and Gender in Research.

Reporting on sex and gender	None
Population characteristics	None
Recruitment	None
Ethics oversight	None

Note that full information on the approval of the study protocol must also be provided in the manuscript.

Field-specific reporting

Please select the one below that is the best fit for your research. If you are not sure, read the appropriate sections before making your selection.

☒ Life sciences ☐ Behavioural & social sciences ☐ Ecological, evolutionary & environmental sciences

For a reference copy of the document with all sections, see nature.com/documents/nr-reporting-summary-flat.pdf

Life sciences study design

All studies must disclose on these points even when the disclosure is negative.

Sample size	Replicates are biologically representing independent experimental subject. Experiments were duplicated at least 3 times and sample size was qualified according to methodology and was specialized in figure legends.
Data exclusions	No data were excluded
Replication	Each experiment was replicated at least 3 times
Randomization	All participants were allocated to groups randomly
Blinding	The investigators were blinded to group allocation during data collection and analysis

Reporting for specific materials, systems and methods

We require information from authors about some types of materials, experimental systems and methods used in many studies. Here, indicate whether each material, system or method listed is relevant to your study. If you are not sure if a list item applies to your research, read the appropriate section before selecting a response.

Materials & experimental systems

n/a	Involved in the study	
☐	☒	Antibodies
☒	☐	Eukaryotic cell lines
☒	☐	Palaeontology and archaeology
☐	☒	Animals and other organisms
☒	☐	Clinical data
☒	☐	Dual use research of concern

Methods

n/a	Involved in the study	
☒	☐	ChIP-seq
☒	☐	Flow cytometry
☒	☐	MRI-based neuroimaging

Antibodies

Antibodies used	Antibody (Dilution, Catalog #, Supplier, are all shown below:<br>Akt (1:1000, 4685, Cell Signaling Technology (CST))<br>p-Akt Ser473 (1:1000, 4060, CST)<br>p-Akt Thr308 (1:1000, 13038S, CST)<br>AMPKα (1:1000, 5832, CST)<br>p-AMPKα Thr172 (1:1000, 2535, CST)<br>mTOR (1:1000, 2983, CST)

	p-mTOR (1:1000, 2976, CST)<br>p-p70 S6K Thr389 (1:1000, 9205, CST)<br>p70 S6K (1:1000, 2708, CST)<br>LEF1 (1:1000, ab85052, Abcam)<br>β-catenin (1:1000, ab32572, Abcam)<br>WE6 (1:5, 348 WE6-s, Developmental Studies Hybridoma Bank)<br>β-actin (1:2000, ab8826, Abcam).<br>BrdU solvent (20 µL of 20 mM solution, B5002, Sigma)<br>β-catenin antibody (1:200, Cat. ab32572, RRID: AB_725966, Abcam)<br>Alexa Fluor 488-conjugated goat anti-rabbit IgG secondary antibody (1:400, Jackson ImmunoResearch, West Grove, PA, USA, Cat.111-545-003, RRID: 334 AB_2338046)<br>DAPI (1:2500, Cat.D9542, Merck, Germany)<br>Anti-BrdU (1:250, ab6326, Abcam, USA)
Validation	For histology, all antibodies were commercially available, tested and validated; in our laboratory, we additionally established staining on control slides of Gecko organs that should express the antigen (immunofluorescence staining and antibody dilution titration on control slides). For all stainings, we used positive control slides (=tissue that should express the antigen) and did negative controls (no primary ab / no secondary ab).<br>For western blot, all antibodies were commercially avaible, tested and validated; in our laboratory, we additionally tested the expression of the antigens in Gecko tissues to determine the suitable dilution, incubation and exploration details.

Animals and other research organisms

Policy information about studies involving animals; ARRIVE guidelines recommended for reporting animal research, and Sex and Gender in Research

Laboratory animals	Adult G. japonicus with intact tails were grouped into control and experimental groups and the average body weight was 4.4 ± 0.495 g.
Wild animals	No wild animals were used in this study.
Reporting on sex	Half male and half female individuals were enrolled in the test.
Field-collected samples	No field collected samples were used in this study.
Ethics oversight	All animal studies were undertaken in accordance with Animal Welfare Guidance of China for the protection of animals used for experimental purposes. All experiments were approved by the Institutional Animal Care and Use Committee of the Nantong University.

Note that full information on the approval of the study protocol must also be provided in the manuscript.
